# Promoter methylation of DNA homologous recombination genes is predictive of the responsiveness to PARP inhibitor treatment in testicular germ cell tumors

**DOI:** 10.1002/1878-0261.12909

**Published:** 2021-03-02

**Authors:** João Lobo, Vera Constâncio, Catarina Guimarães‐Teixeira, Pedro Leite‐Silva, Vera Miranda‐Gonçalves, José Pedro Sequeira, Laura Pistoni, Rita Guimarães, Mariana Cantante, Isaac Braga, Joaquina Maurício, Leendert H. J. Looijenga, Rui Henrique, Carmen Jerónimo

**Affiliations:** ^1^ Cancer Biology and Epigenetics Group IPO Porto Research Center (GEBC CI‐IPOP) Portuguese Oncology Institute of Porto (IPO Porto) & Porto Comprehensive Cancer Center (P CCC) Portugal; ^2^ Department of Pathology Portuguese Oncology Institute of Porto (IPOP) Portugal; ^3^ Department of Pathology and Molecular Immunology Institute of Biomedical Sciences Abel Salazar University of Porto (ICBAS‐UP) Portugal; ^4^ Princess Máxima Center for Pediatric Oncology Utrecht The Netherlands; ^5^ Department of Biology University of Pisa Italy; ^6^ Department of Urology Portuguese Oncology Institute of Porto (IPOP) Portugal; ^7^ Department of Medical Oncology Portuguese Oncology Institute of Porto (IPOP) Portugal

**Keywords:** DNA methylation, DNA repair, homologous recombination, Olaparib, PARP inhibitors, testicular germ cell tumors

## Abstract

Testicular germ cell tumors (TGCTs) are the most common cancers in men aged 15–39 years and are divided into two major groups, seminomas and nonseminomas. Novel treatment options are required for these patients, to limit side effects of chemotherapy. We hypothesized that promoter methylation of relevant homologous recombination (HR) genes might be predictive of response to poly‐ADP ribose polymerase inhibitors (PARPis) in TGCTs. We report a study pipeline combining *in silico*, *in vitro,* and clinical steps. By using several databases and *in silico* tools, we identified *BRCA1*, *RAD51C*, *PALB2*, *RAD54B,* and *SYCP3* as the most relevant genes for further investigation and pinpointed specific CpG sites with pronounced negative correlation to gene expression. Nonseminomas displayed significantly higher methylation levels for all target genes, where increased methylation was observed in patients with more differentiated subtypes and higher disease burden. We independently performed second‐line targeted validation in tissue series from TGCT patients. A moderate and/or strong anti‐correlation between gene expression (assessed by RNA‐sequencing) and promoter methylation (assessed by 450k array) was found, for all of the targets. As a proof of concept, we demonstrated the sensitivity of TGCT cell lines to Olaparib, which associated with differential methylation levels of a subset of targets, namely *BRCA1* and *RAD51C*. Our findings support the use of HR genes promoter methylation as a predictor of the therapeutic response to PARPis in patients with TGCT.

## Graphical abstract

We report a study pipeline for exploring whether aberrant promoter methylation of genes involved in the homologous recombination pathway determines responsiveness to PARP inhibitors in testicular germ cell tumor patients. *In silico* analyses were followed by validation using quantitative methylation‐specific PCR. We showed differential response of germ cell tumor cell lines to Olaparib, associated with *BRCA1*/*RAD51C* promoter methylation.

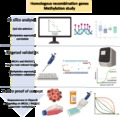

**Abbreviations**
AJCC: American Joint Committee on Cancer
BER: base‐excision repair
CH: choriocarcinoma
DMSO: dimethyl sulfoxide
EC: embryonal carcinoma
GCNIS: germ cell neoplasia in situ
HR: homologous recombination
NS: nonseminoma
PARPi: poly‐ADP ribose polymerase inhibitor
qMSP: quantitative methylation‐specific real‐time PCR
RT‐qPCR: real‐time quantitative polymerase chain reaction
SE: seminoma
SMART: Shiny Methylation Analysis Resource Tool
TCGA: The Cancer Genome Atlas
TE: teratoma
TGCT: testicular germ cell tumor
TSS: transcription starting site
YST: yolk sac tumor

## 1 Introduction

Testicular germ cell tumors (TGCTs) are among the most frequent solid neoplasms in young‐adult Caucasian men, and incidence is increasing worldwide due to life‐style changes [1]. These patients show an overall good prognosis (especially due to high efficacy of platinum‐based chemotherapy) and often become long‐term cancer survivors [2]. Nonetheless, a substantial number of patients are overtreated, receiving adjuvant treatment although they would never endure disease recurrence. Consequently, increased incidence of side effects has been observed (secondary neoplasms, metabolic syndrome, coronary heart disease and others) in those young patients [3]. Thus, there is a need for biomarkers that reliably stratify patients concerning the risk of relapse [4]. Additionally, there is an urgent need for novel and less toxic targeted treatment options that might allow for dose reduction of chemotherapy agents. Such therapies may well be key for targeting patients developing cisplatin resistance, who display poor prognosis and eventually die of disease [5, 6].

Type II TGCTs (by far the most common germ cell tumors) derive from a common precursor, germ cell neoplasia *in situ* (GCNIS), and their genesis is related to a ‘genvironmental model’, in which genetic, environmental and epigenetic events contribute to tumorigenesis [7]. They are divided into seminomas (SEs) and nonseminomas (NSs), with different clinical behavior, the latter comprising several subtypes [embryonal carcinoma (EC), yolk sac tumor (YST), choriocarcinoma (CH), teratoma (TE) and mixtures of two or more components, the mixed tumors] [8]. The epigenetic landscape of these tumors differs markedly and resembles the corresponding cell of origin during embryonic and germ cell development [9]. Hence, epigenetic events are promising disease biomarkers [10].

DNA methylation is by far the most well‐studied epigenetic mechanism of gene expression regulation. Specific gene promoter methylation targets are increasingly being used as biomarkers for diagnosis, risk stratification, follow‐up, and response to therapies in cancer, both in tissues and in liquid biopsies [11, 12]. In an integrated analysis, Shen *et al*. [13] reported that promoter methylation of homologous recombination (HR) DNA repair genes *BRCA1* and *RAD51C* were among the most commonly hypermethylated loci in TGCTs, suggesting its use as biomarkers of response to poly‐ADP ribose polymerase inhibitors (PARPis). Indeed, an *in vitro* study showed that TGCT cell lines are sensitive to Olaparib [14], and clinical trials are currently underway for assessing the efficacy of these drugs in TGCT patients [15].

Herein, we aimed to further explore this hypothesis in a well‐established TGCT patient cohort and representative TGCT cell lines. We make use of several *in silico* tools to interrogate the most relevant CpG sites regulating expression of various HR genes, validate methylation and expression data by exploring online available databases and further confirm the findings in our own tissue cohort. Moreover, we show that TGCT cell lines are indeed sensitive to Olaparib and we correlate this effect with HR genes' methylation levels.

## 2 Methods

### 2.1 *In silico* analyses: selection of most relevant CpG sites and targets

To interrogate specific CpG sites within the promoter region of genes involved in HR DNA repair pathway, TCGA Human methylation 450k array data were retrieved from Shiny Methylation Analysis Resource Tool (SMART) App website http://www.bioinfo‐zs.com/smartapp/ [16]. A list of total number of CpG probes analyzed in Human methylation 450k within the following genes was obtained: *ATM, BARD1, BLM, BRCA1, BRCA2, BRCC3, BRIP1, EME1, FANCD2, MUS81, NBN, PALB2, RAD51C, RAD51D, RAD52, RAD54D, RBBP8, RPA1, RPA2, SSBP1, SYCP3, UIMC1*. These data were merged with that from HumanMethylation 450 v1.2 Manifest File (Illumina, Diego, CA, USA), and CpG probes were filtered using UCSC CpG Island (Island) and UCSC RefGene group (TSS200 or TSS1500) to ascertain the number of CpGs located concomitantly in CpG islands and promoter regions (defined as 1500 bp upstream the transcription starting site, TSS). All data were processed in rstudio 1.2.5001 for macos software(RStudio, Boston, MA, USA). Precise localization of CpGs was recorded for guiding design of primers and probes (see below).

Because we aimed to ascertain CpG sites within the gene promoter in which methylation levels inversely correlated with gene expression levels (i.e., biological meaningful methylated CpG sites, that influence gene expression), we used SMART App website [16] to perform pair‐wise correlation analyses of DNA methylation‐gene expression using the following criteria: Dataset—TGCT dataset; Methylation value—Beta‐value; Gene expression—Log2‐scaled(TPM + 1) values; Correlation coefficient—Spearman; and Aggregation Method—mean. Then, the most significant and highly anti‐correlated genes/CpG sites (higher correlation coefficients) were selected for validation by targeted analyses using qMSP (see below).

### 2.2 *In silico* analyses: exploring selected targets within The Cancer Genome Atlas (TCGA) cohort

The selected genes and specific promoter regions were firstly investigated by *in silico* analysis of TCGA dataset. Mean aggregation Human Methylation 450k (beta‐values) (of the significant CpGs) and RNA‐seq (Log2‐scaled(TPM + 1) values) data were imported from SMART App website [16] and processed and analyzed in spss 25.0 for macos software (IBM, Armonk, NY, USA). Clinical data of the 133 TGCT patients included in the database were imported from cBioPortal for Cancer Genomics (https://www.cbioportal.org/) [17] and Firehose Broad GDAC TGCT Clinical Archives (https://gdac.broadinstitute.org/). Gene expression of *SYCP3* across normal tissue samples of several organs and TGCT samples was extracted from GEPIA (http://gepia.cancer‐pku.cn/index.html) [18], which includes data from GTExPortal (https://gtexportal.org/home/) [19]. Protein expression in normal tissues was extracted from The Human Protein Atlas (https://www.proteinatlas.org/) [20].

### 2.3 Patient samples

A retrospective cohort of type II TGCT patients was selected for this study to validate *in silico* findings. Patients underwent radical inguinal orchiectomy between 2005 and 2018 at Portuguese Oncology Institute of Porto (IPO Porto), Portugal. A total of 150 TGCT patients were included, all treated by the same multidisciplinary team. Specimens were routinely fixed in formalin and paraffin‐embedded for subsequent histological examination. Importantly, all histological material was re‐classified by the same TGCT‐dedicated Pathologist according to the most recent 2016 World Health Organization classification (full cohort reported in Ref. [21]). Clinical information was also reviewed, and patients were staged according to the most recent American Joint Committee on Cancer (AJCC) 8th edition. Patients presenting metastases at diagnosis were further categorized following the International Germ Cell Cancer Collaborative Group (IGCCCG) prognostic system, as recommended [22, 23]. Follow‐up was last updated in May 2019.

A representative tumor block, with > 70% tumor cellularity and low necrosis content, was selected by a TGCT‐dedicated Pathologist. Importantly, within the 57 Mixed Tumors, tumor components were individually dissected and independently considered for DNA extraction (as previously reported by us [24]). Thus, a total of 238 individual tumor samples were included and 8‐ and 3‐μm‐thick sections were ordered for DNA extraction and for immunohistochemistry, respectively.

A summary of the study cohort is depicted in Table S1. This study was approved by the Ethics Committee of IPO Porto (CES‐IPO‐1/2018). All procedures performed in tasks involving human participants were in accordance with the ethical standards of the institutional and/or national research committee and with the 1964 Helsinki declaration and its later amendments or comparable ethical standards.

### 2.4 Immunohistochemistry and immunocytochemistry

Immunohistochemistry was performed in representative slides available from 96 patients of the same patient cohort described above. The immunohistochemistry protocol is described in full in [25]. For immunocytochemistry, the four cell lines used in the study (see below) were plated in 24‐well plates at density of 20 000 cells/well and incubated overnight. Cells were washed with PBS and fixed with 4% paraformaldehyde (Santa Cruz, USA) for 15 min, followed by permeabilization with Triton X‐100 0.25% in 1×PBS for another 15 min. Then, the same protocol described for immunohistochemistry was performed. Slides were incubated for 1 h with the primary antibody anti‐SYCP3 (n. HPA039635, polyclonal, 1 : 500), at room temperature. Normal testis with preserved spermatogenesis (Johnsen's score 10) was used as external positive control in each run; entrapped seminiferous tubules within tumor tissue additionally served as internal positive controls of the staining. Negative controls, consisting of omission of primary antibodies, were included per run.

Immunoexpression was independently assessed for each TGCT component (i.e., independently for each histological component within mixed tumors). Thus, a total of 141 TGCT components were scored. Both intensity and percentage of positive cells were assessed; intensity of staining was classified as ‘weak = 1’, ‘moderate = 2’ and ‘strong = 3’ as previously defined [26]; percentage of positive cells was considered with 10% intervals, and categorized as ‘negative = 0’, ‘< 10% = 1’ and ‘≥ 10% = 2’. A final combined score (intensity × percentage of stained cells) was computed, and categorized as ‘negative = 0’, ‘low = 1’ and ‘high = 2–4’.

### 2.5 Cell lines, Olaparib treatment, cell viability, and colony formation assays

TGCT cell lines (*n* = 4) including TCam‐2 (a SE‐like cell line) and NCCIT, 2102Ep, and NTera‐2 (representative of NS) were kindly provided by L. Looijenga. Cell lines have been previously characterized, including copy number alterations, and have been authenticated via STR profiling, with profiles compared to the database https://www.lgcstandards‐atcc.org/en.aspx and to the ones available in previous publications [27, 28]. Cells were cultured as described elsewhere [29], maintained in low passages, and tested negative for *Mycoplasma* spp. (Clontech Laboratories, Mountain View, CA, USA; tested twice a month). Olaparib (AZD2281) was purchased from Selleckchem (Catalog No. S1060; Houston, TX, USA) in a stock solution (10 mm) already dissolved in dimethyl sulfoxide (DMSO).

Cell viability assay was performed as described [30] at 24, 48, 72, and 96 h of treatment with Olaparib, using dosages in the approximate same range as in the study of Cavallo *et al*. [14], ranging from 100 nm to 3 µm. These doses are way below the tolerated doses used in the clinical setting (18 µm), and within doses previously shown to produce clinical benefit in the clinic, as mentioned in [31, 32, 33, 34]. Briefly, cells were plated into 96‐well plates in medium at density of 6000 cells/well for NCCIT, NTera‐2 and 2102Ep and 4000 cells/well for TCam‐2 (seeding densities previously optimized) and incubated overnight, at 37 °C in 5% CO_2_. The vehicle alone (DMSO in medium, 1%) was included in each experiment. Resazurin (Canvax Biotech, Córdoba, Spain) was used for the viability assay. The culture medium was removed, and cells were incubated during 3 h at 37 °C with 100 μL of 1 : 10 Resazurin solution in culture medium. The solution was then removed, and spectrophotometric measurement was performed at 560 nm (reference wavelength: 600 nm) in a microplate reader (FLUOstar Omega, BMG Labtech, Ortenberg, Baden‐Wuerttember, Germany). Wells with the Resazurin solution were used as blank to correct OD values. ODs obtained for each time point were all normalized for the 0 h‐time point. Olaparib and vehicle were freshly added to the wells at each time point, and the procedure was repeated the next day. All experiments were performed with biological triplicates, each with experimental triplicates. IC50 values were extrapolated from the sigmoidal dose‐response (four‐parametric logistic equation) with variable slope, as calculated on graphpad prism 6 (GraphPad Software, La Jolla, CA, USA).

For the colony formation assay, the four cell lines were seeded in 6‐well culture plates at the following densities (2000 cells/well for NCCIT, NTera‐2 and 2102Ep cell lines and 3000 cells/well for TCam‐2 cell line). Cells were treated with 500 nm, 1 µm, and 3 µm Olaparib for 7 days with drug renewal every 48 h. Colonies were fixed in methanol during 30 min and stained with Diff‐Quik method. Colonies depicting more than 50 single cells were counted in six replicates of every experimental condition, by two researchers. Results were represented as survival fraction (SF) according to the following formula SF = [number of colonies/(number of plated cells × platting efficiency)] and plotted in graphpad prism 6.

### 2.6 DNA extraction and bisulfite treatment

DNA was extracted from cell lines using phenol–chloroform method and from tumor tissues using RNA/DNA Purification Plus Kit (Norgen Biotek, Thorold, Canada), according to manufacturers' instructions. Genomic DNA was quantified in NanoDrop™ Lite Spectophotometer (Cat. ND‐LITE; Thermo Scientific™, Waltham, MA, USA), and 1000 ng was bisulfite‐treated using the EZ‐96 DNA Methylation‐Gold Kit (Zymo Research, Irvine, CA, USA), according to manufacturers' protocol.

### 2.7 Primer/probe design and quantitative methylation‐specific real‐time PCR (qMSP)

Genomic DNA of the target genes (plus 1500 bp upstream) was acquired from UCSC Genome Browser on Human Dec. 2013 (GRCh38/hg38) Assembly. Bisulfite‐treated methylated DNA sequence was obtained through Methyl Primer Express v1.0. Specific forward/reverse primers and probes were designed to accommodate the CpG sites relevant for the study (as determined by our *in silico* analyses, see above). Full details about the primer/probe design and amplicons studied are provided in File S1. Importantly, to assure optimal primer/probe properties, sequences were analyzed using the Primer Express 3.0—Primer Probe Test Tool, and additionally with the Beacon Designer, Premier Biosoft (Palo Alto, CA, USA). Finally, to assure specificity for only one (specific) PCR product, primer sequences were run through Bisearch Primer Design and Search Tool [35].

qMSP reactions were run in 384‐well plates (45 cycles) in QuantStudio 12K Flex Real‐Time PCR System (Thermo Fisher, Foster, CA, USA). In brief, 1 µL bisulfite‐treated DNA, 5 µL MasterMix Xpert Fast Probe (GRISP) with ROX, a variable volume of primers and probe at 10 μm and sterile bi‐distilled water in a total volume of 10 µL were added to each well (Table S2). A multiplex qMSP reaction was run, consisting of ‘panel #1’ (*BRCA1*, *PALB2* and *RAD54B*) and ‘panel #2’ (*RAD51C* and *ACTB*); *SYCP3* was run in singleplex reaction. Results were normalized to *ACTB* as previously described [36]. For cell lines, five biological replicates were included, each with technical triplicates. For tissues, all reactions were run in triplicates. Three no template controls (NTC) and two negative controls (Human HCT116 DKO Non‐Methylated DNA, D5014‐1; Zymo) were included in every plate, assuring absence of contaminations and specificity for the methylated DNA template. Serial dilutions (five, in duplicate) of Human HCT116 DKO Methylated DNA (D5014‐2; Zymo) were included in each plate, used to compute a standard curve and assured run efficiency and interpolate comparability. Results were plotted as relative methylation levels (Target gene/*ACTB*), multiplied by 1000 for easier tabulation.

### 2.8 RNA extraction, cDNA synthesis, and real‐time quantitative PCR (RT‐qPCR)

RNA extraction and cDNA synthesis were performed as reported in Ref. [37]. RT‐qPCR reactions were run on the same format and platform described above, with specific primers for *BRCA1* and *RAD51C* already reported as relevant in the study on PARPis of Kounatidou *et al*. [38] (run conditions reported in Table S2) and 5 µL of Xpert Fast SYBER Mastermix Blue (GRiSP Research Solutions, Porto, Portugal). Serial dilutions of cDNA obtained from Human Reference Total RNA (Cat. 750500; Agilent Technologies^®^, Santa Clara, CA, USA) were used to compute standard curves for each plate. All experiments were run in triplicate, and two negative controls were included in each plate. Relative expression of target genes was normalized to housekeeping GUSB, determined as: [Gene Expression Level = (Gene Mean Quantity/GUSB Mean Quantity) × 1000].

### 2.9 Statistical analysis

Data were tabulated using Microsoft Excel 2016 and analyzed and plotted using graphpad prism 6 and ibm spss statistics version 24 (IBM, Armonk, NY, USA). Percentages were calculated based on the number of cases with available data. Nonparametric (Mann–Whitney and Kruskal–Wallis) tests were used for comparing distribution of continuous variables among groups, as appropriate. Nonparametric Spearman correlation test was used to assess correlations between continuous variables and interpretation of strength of results was done as described by Evans [39]. All *P*‐values were adjusted for multiple comparisons (Dunn's test and Bonferroni correction, as appropriate). Chi‐square and Fisher exact test were used as necessary for establishing associations between categorical variables. Statistical significance was set at *P* < 0.05 and is reported in graphs as follows: *P < 0.05; **P < 0.01; ***P < 0.001; ****P < 0.0001.

## 3 Results

### 3.1 *In silico* CpG selection

**Fig. 1:**
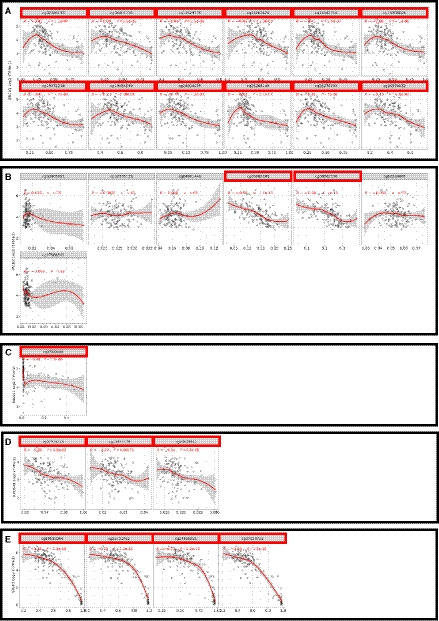
*In silico* CpG selection based on individual expression‐methylation anti‐correlations. A—BRCA1; B—PALB2; C—RAD51C; D—RAD54B; E—SYCP3. Methylation levels are reported as beta‐values (450k array) and gene expression as Log2‐scaled (TPM + 1) (RNA‐sequencing) values. The line and shaded gray area derive from Spearman correlation and were retrieved as modeled and defined by SMART app (see appropriate reference in text for more details). Analyses include *n* = 136 TGCTs. TPM, transcript per million.

*In silico* analyses of CpG sites of the referred genes are summarized in File S2. The total number of CpG sites included in the 450k methylation array for each gene was ascertained. Because we wanted to focus on CpGs most related to gene expression regulation, the CpG sites within CpG islands and located within the gene promoter were filtered (as defined above). Finally, the significance of anti‐correlation between methylation and transcript levels was determined for every individual CpG site and for the CpGs' aggregation. Of all genes, *BRCA1* was the one with most CpG sites detaining significant anti‐correlation between gene expression and methylation (*n* = 12). From correlation coefficients' analysis, the strongest anti‐correlations were found for *BRCA1*, *PALB2*, *RAD51C*, *RAD54B,* and *SYCP3* (Fig. 1A–E). The strongest anti‐correlation was observed for *SYCP3* (*r* = −0.73 to −0.74). Based on these assessments, these five genes were selected for further validation in subsequent analyses. Bar graph representation illustrative of gene expression versus methylation of individual CpGs is provided in Figs [Link], [Link], [Link].

### 3.2 *In silico* analyses of gene promoter methylation and gene expression

**Fig. 2:**
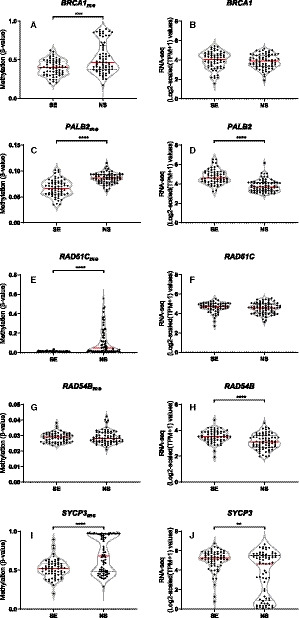
Methylation and expression levels of target genes. BRCA1 (A, B), PALB2 (C, D), RAD51C (E, F), RAD54B (G, H), and SYCP3 (I, J) methylation/expression levels among seminomas and nonseminomas. Methylation levels are reported as beta‐values (450k array) and gene expression as Log2‐scaled (TPM + 1) (RNA‐sequencing) values. Error bars indicate median and interquartile range. Statistical test was Mann–Whitney. Analyses include *n* = 63 seminomas and *n* = 70 nonseminomas. See text for details. SE, seminoma; NS, nonseminoma; TPM, transcript per million.

We then explored in more detail both the gene promoter methylation (using mean aggregation of significantly anti‐correlated CpG sites depicted above) and transcript levels of selected genes in the TCGA‐TGCT cohort. Methylation levels were significantly higher in NS compared to SEs for *BRCA1* (*P* = 0.0005), *PALB2* (*P* < 0.0001), *RAD51C* (*P* < 0.0001) and *SYCP3* (*P* < 0.0001) (Fig. 2). Interestingly, absolute methylation levels (beta‐values) were in the high range for *BRCA1* and *SYCP3*, but within the lower range for *PALB2*, *RAD51C,* and *RAD54B*. As for expression levels, in accordance with methylation findings, significantly lower expression was found in NS compared to SE for *PALB2* (*P* < 0.0001), *RAD54B* (*P* < 0.0001) and *SYCP3* (*P* = 0.0023). When discriminating among tumor subtypes (illustrated in Fig. S4), higher methylation levels were found in Mixed Tumors, YST, and TE and, as expected, these were the subtypes with lowest expression levels.

**Fig. 3:**
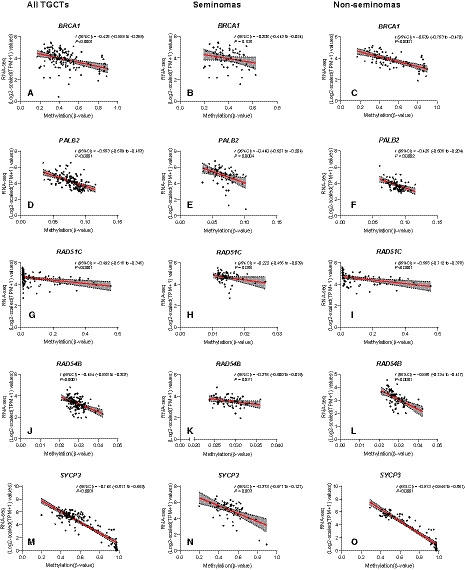
Expression‐methylation anti‐correlation analyses. Anti‐correlation analyses for BRCA1 (A–C), PALB2 (D–F), RAD51C (G–I), RAD54B (J–L), and SYCP3 (M–O) in all testicular germ cell tumors, and individually for seminomas and nonseminomas. The aggregation of significant CpG sites identified is considered. Methylation levels are reported as beta‐values (450k array) and gene expression as Log2‐scaled (TPM + 1) (RNA‐sequencing) values. The line was modeled through simple linear regression, and the statistical test was Spearman correlation test. Shaded gray area relates to error, set to 95% confidence. Analyses include *n* = 133 TGCTs (*n* = 63 seminomas and *n* = 70 nonseminomas). See text for details. TGCTs, testicular germ cell tumors; TPM, transcript per million.

Regarding correlation analyses, all selected genes exhibited moderate/strong inverse correlations between methylation and expression levels (*r* = −0.425, −0.583, −0.492, −0.454 and −0.748 for *BRCA1*, *PALB2*, *RAD51C*, *RAD54B,* and *SYCP3*, respectively). Importantly, correlations either lost significance or strength when considering only SEs (except for *PALB2*), whereas enrichment was displayed by NS tumors (Fig. 3). Moreover, anti‐correlations were maintained when stratified analysis per NS histology was performed (in categories with sufficient number of patient samples, Table S3): when considering only pure ECs or only Mixed Tumors, the significant anti‐correlation between methylation and expression was maintained for all genes (except for *PALB2* in pure ECs, which did not achieve significance).

Concerning clinicopathological correlates, lower *RAD51C* and *RAD54B* expression levels significantly associated with nodal (N+) disease (*P* = 0.0220 and *P* = 0.0137, respectively), and significantly higher *RAD51C* methylation levels were found in metastatic (M+) disease (*P* = 0.0338) (Fig. S5). Additionally, when performing analyses stratified by histology, lower expression of *RAD51C* in N+ disease compared to N0 disease was maintained for Mixed Tumors (*P* = 0.007) and for NSs as a whole (*P* = 0.044), but without significant differences regarding methylation. Additionally, we found statistically significant higher methylation of *RAD54B* in M+ disease compared to M0 within this histological category (*P* = 0.049), and also when considering all NS subtypes (*P* = 0.012). No other significant associations were found. Furthermore, no significant correlations were found between patients' age and gene promoter methylation or gene expression levels, including when SEs and NSs were analyzed separately, indicating that patients' age is not biasing the results.

### 3.3 Validation in independent tissue cohort of TGCT patients

**Fig. 4:**
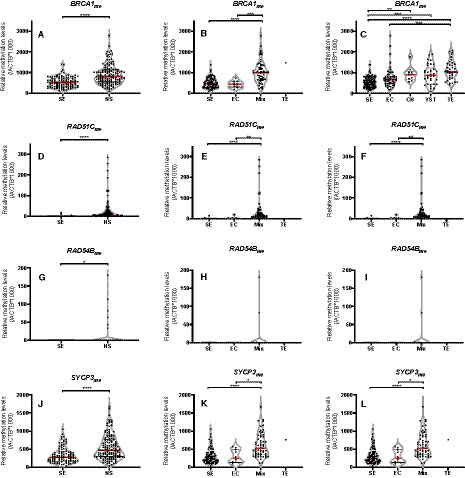
Differential methylation levels (qMSP validation) of gene targets. Differential methylation levels of BRCA1 (A–C), RAD51C (D–F), RAD54B (G–I), and SYCP3 (J–L) between seminoma and nonseminoma tumor components, between patient histological categories (including mixed tumors as a whole), and among individually dissected tumor subtypes. Results are normalized to ACTB. Error bars indicate median and interquartile range. Statistical test was Mann–Whitney/Kruskal–Wallis. Analyses include *n* = 103 seminoma and *n* = 135 nonseminoma tumor components (left); *n* = 82 pure seminomas, *n* = 10 pure embryonal carcinomas, *n* = 57 mixed tumors, and *n* = 1 pure teratoma (center); and *n* = 103 seminoma, *n* = 54 embryonal carcinoma, *n* = 10 choriocarcinoma, *n* = 34 yolk sac tumor, and *n* = 37 teratoma components (right). SE, seminoma; NS, nonseminoma; EC, embryonal carcinoma; YST, yolk sac tumor; CH, choriocarcinoma; TE, teratoma.

We then attempted to validate these findings in our own independent and well‐established TGCT cohort. Concordantly, we also found that NSs exhibited significantly higher BRCA1 (*P* < 0.0001), RAD51C (*P* < 0.0001), RAD54B (*P* = 0.0123) and SYCP3 (*P* < 0.0001) methylation levels compared to SEs (Fig. 4, left column). When we grouped patients into individual histologies (in an approach matching the methodology in TCGA cohort), the results paralleled the *in silico* analyses, being most remarkable (and statistically significant differences) among SEs and mixed tumors (Fig. 4, middle column). This also disclosed significantly higher methylation levels for mixed tumors compared to pure ECs. Within the 238 TGCT components analyzed, some degree of methylation could be detected in 238 (100%), 98 (41.2%), 8 (3.4%), and 233 (97.9%) samples for *BRCA1*, *RAD51C*, *RAD54B,* and *SYCP3*, respectively. *PALB2* promoter did not display methylation in our cohort (in consonance with the low range of beta‐values in the *in silico* analyses). When breaking up individual tumor subtypes among mixed tumors, again SEs disclosed significantly lower methylation levels, whereas the highest levels were exhibited by YST and TE (Fig. 4, right column).

Then, we focused on assessing *BRCA1* and *RAD51C* expression levels in our tissue cohort. We found seminomas to have significantly higher expression of both genes compared to NSs (*P* < 0.0001 for both, Fig. S6A,B). When exploring individual tumor subtypes, the more differentiated subtypes (choriocarcinoma, yolk sac tumor and teratoma) showed significantly lower expression of these targets (Fig. 6C,D). Importantly, the pattern of expression of *BRCA1* and *RAD51C* now shown by RT‐qPCR (not so evident by RNA‐sequencing in the *in silico* analysis, with limited numbers of the more differentiated histologies) completely opposes the methylation data (in both cohorts, ours and TCGA's). In fact, like for *in silico* analyses, we performed anti‐correlation analyses in our cohort, and found BRCA1 and RAD51C methylation/expression to be significantly anti‐correlated (*P* = 0.0009 and *P* < 0.0001, Fig. 6E,F).

Regarding clinicopathological correlates, significantly higher *BRCA1* methylation levels were found in stage II/III disease compared to stage I disease (*P* = 0.0423) and significantly higher *RAD51C* methylation levels where depicted by IGCCCG intermediate/poor prognosis disease compared to good prognosis disease (*P* = 0.0096) (Fig. S7). No additional significant associations with clinical traits were found. Methylation levels of each target did not significantly associate with patients' age, either for SE or NS patients.

Finally, given the very high anti‐correlation observed for *SYCP3* gene expression‐methylation and the high frequency of *SYCP3* methylation observed (98% of our samples), SYCP3 protein expression was explored in more detail. *SYCP3* is a meiosis‐specific gene, but also expressed in several cancer types, in which it impairs mitotic recombination by interfering with *BRCA2*, sensitizing certain tumor cells to PARPis [40]; hence, we wanted to investigate this in TGCTs as well. *In silico* analysis of public databases confirmed *SYCP3* overexpression in normal testis (versus other tissues/organs), both at RNA and protein levels, and also in TGCTs compared to other cancer types (Fig. S8). We then validated this at the protein level by immunohistochemistry in our tissue set (illustrative examples in Fig. S9). SYCP3 was, indeed, intensely positive in normal testis tissues (staining nucleus of spermatocytes), whereas in TGCTs, immunoexpression was observed in the lower range, with most individual tumor components being negative (*n* = 85) or disclosing < 10% weakly positive tumor cells dispersed throughout the tumor bulk (*n* = 30). In our analyses, NSs depicted significantly lower immunoexpression score compared to SEs (*P* < 0.0001), also when discriminating for subtypes, paralleling transcript *in silico* findings. However, we were not able to detect positivity for this marker in the four TGCT cell lines.

### 3.4 Methylation of selected genes and sensitivity to PARPi Olaparib

**Fig. 5:**
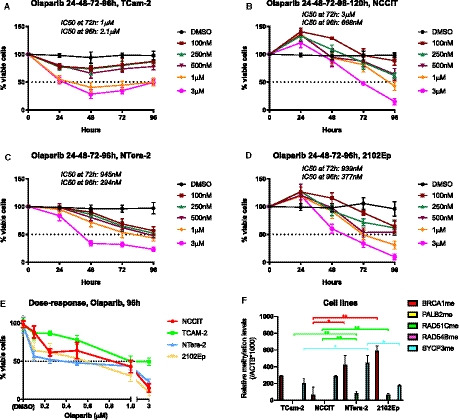
Sensitivity to Olaparib in TGCT cell lines in respect to methylation of target genes. (A–D) Cell viability curves for TCam‐2, NCCIT, NTera‐2, and 2102Ep; (E) combined dose‐response curves for all four cell lines; (F) differential methylation levels of selected target genes among the different cell lines. Results are normalized to ACTB. Error bars indicate mean and standard deviation. Statistical test was Kruskal–Wallis with Dunn's test for multiple comparisons. Three biological replicates were included in Olaparib viability experiment, and five biological replicates in qMSP, both with experimental triplicates.

**Fig. 6:**
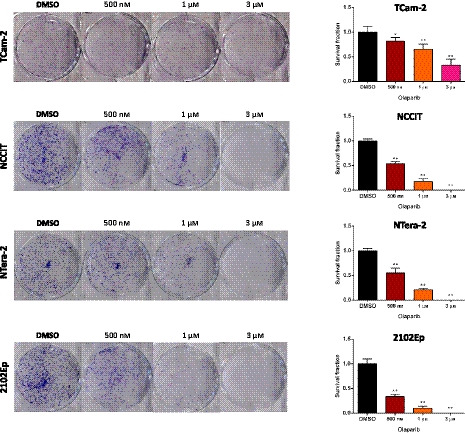
Colony formation assay in all four cell lines upon treatment with Olaparib. Photographs of the effect on colony number and graphical representation of survival fraction upon treatment with Olaparib, compared to control condition, for (A) TCam‐2, (B) NCCIT, (C) NTera‐2, and (D) 2102Ep. Error bars indicate mean and standard deviation. Statistical test was Kruskal–Wallis with Dunn's test for multiple comparisons. Six biological replicates were included.

Olaparib significantly reduced cell viability in all cell lines (Fig. 5). TCam‐2 (a SE‐like cell line) was the least sensitive, with an IC50 at 72 h of 1 µm, and cells actually recovering at 96 h, showing an IC50 of 2.1 µm (Fig. 5A). Contrarily, NS cell lines were the most sensitive to the compound. For NCCIT, a reduction in cell viability was only apparent beyond 24 h of drug exposure (as for 2102Ep), with cells disclosing an IC50 of 3 µm at 72 h and of 668 nm at 96 h of exposure (Fig. 5B). The most sensitive cell line was NTera‐2 (IC50 at 72 and 96 h of 945 and 294 nm, respectively), followed by 2102Ep (IC50 at 72 and 96 h of 939 and 377 nm, respectively) (Fig. 5C,D). Dose‐response curves at 96 h of exposure are depicted in Fig. 5E, for comparability of all four cell lines. Regarding methylation analyses, whereas *PALB2* or *RAD54B* methylation was absent in cell lines (compatible with tissue findings, see above), both *BRCA1*, *RAD51C* and *SYCP3* were differentially methylated in the studied cell lines. Importantly, *RAD51C* methylation was found only in the two most sensitive cell lines (NTera‐2 and 2102Ep), which also depicted the highest *BRCA1* methylation levels. The least responsive cell lines (NCCIT and TCam‐2) disclosed the lowest *BRCA1* methylation levels. NTera‐2 displayed the highest *SYCP3* methylation levels, whereas 2102Ep depicted the lowest (detailed statistical significance is illustrated in Fig. 5F). These data are in line with our hypothesis, which is further reinforced by expression data (Fig. S10), opposing the methylation profile also in cell lines; the cell line least responsive to Olaparib (TCam‐2) presented the highest *BRCA1* and *RAD51C* expression levels. Also, the most responsive cell lines (NTera‐2 and 2102Ep) showed significantly lower expression levels of both targets. Finally, the colony formation assay further confirmed the differential effect of Olaparib in TCam‐2 compared to other cell lines representative of NS, with greatest impact in colony formation in the latter (Fig. 6).

## 4 Discussion

TGCTs are heterogeneous neoplasms, with diverse histological subtypes and clinical behavior. Treatment guidelines differ for SE and NS patients and according to disease burden/stage [41]. The field of biomarkers for risk stratification of stage I disease is expanding, in an attempt to safely place patients on surveillance and avoid overtreatment, only selecting for adjuvant chemotherapy a subgroup of higher risk patients [42, 43]. Although the currently available serum tumor markers are useful [44], they are dependent on histology and show important limitations [45]. Therefore, epigenetic biomarkers are attractive as diagnostic, prognostic and follow‐up strategies, especially given the improvement of methods of detection in liquid biopsies [46]. Indeed, in TGCTs, the most promising noninvasive biomarker—circulating miR‐371a‐3p—is epigenetic‐based, with compelling evidence of its value for detecting all TGCT subtypes (except mature TE), providing an accurate means of follow‐up and detection of residual disease after chemotherapy [47, 48, 49] (for a recent review see [50]). Since DNA methylation is the most studied epigenetic mechanism, methylation‐based biomarkers are also attractive, particularly in TGCTs, as methylation is involved in germ cell tumors and germ cell development [51], and its landscape is variable across tumor subtypes [52]. For instance, DNA methylation panels including several gene promoters (*RASSF1A*, *CRIPTO*, *HOXA9*, *MGMT,* and *SCGB3A1*) were reported as useful for subtyping and prognostication of disease, including in tissues and liquid biopsies [24, 53, 54], and demethylating agents demonstrated anti‐tumor effects in pre‐clinical studies [55, 56, 57, 58].

Following the report of frequent *BRCA1* and *RAD51C* promoter methylation in TGCTs detected through DNA methylation array (450k) by Shen *et al*. [13], we aimed to study in more detail the HR DNA repair pathway in these tumors. This family of genes is involved in repair of double‐strand breaks following inter‐strand cross‐links, and was previously reported to be impaired in TGCTs [59]. Important work by Cavallo *et al*. [14] indicated that the high sensitivity of EC‐cell lines to cisplatin (which causes the aforementioned double‐strand breaks) was related to reduced proficiency of this pathway; these cell lines showed reduced ability to repair such breaks, reduced RAD51 foci formation and were sensitive to Olaparib monotherapy, in a way dependent of the degree of impairment of HR pathway (and also dependent on *PARP1* expression, differential among TGCT subtypes [60]). This illustrates the mechanism of so‐called synthetic lethality (by simultaneous abrogation of HR and base‐excision repair—BER—pathways), the base of approval of PARPis for BRCA‐mutated breast, ovarian and pancreatic cancer [61]. Olaparib, included in a clinical trial enrolling TGCT patients [15], also potentiated response to cisplatin, substantiating the relevance of characterizing this pathway for predicting response to therapy. These studies provided the rationale for our work, in which we sought to investigate this pathway impairment specifically by promoter methylation of its most relevant members.

**Fig. 7:**
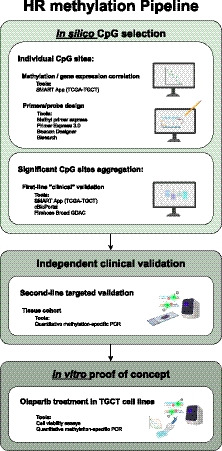
Workflow of the study. The pipeline started with careful *in silico* analyses for achieving the most relevant gene targets within the homologous recombination family of genes and, importantly, the most important individual CpG sites influencing gene expression. These analyses guided primer/probe design, which were optimized also using *in silico* tools. This was followed by validation in our independent set of TGCT tissues, also including expression analyses for the two most remarkable genes of the set, *BRCA1* and *RAD51C*. Finally, as a proof of concept, we performed *in vitro* studies (cell viability and colony formation assay), demonstrating differential sensitivity of the various cell lines related to promoter methylation/expression of target genes. See text for details.

To achieve our goal, we designed a specific pipeline of analysis (Fig. 7). First, we made use of publicly available databases for querying methylation levels of available CpG sites within the promoter of genes related to the HR pathway. Importantly, we focused our analysis on CpGs contained specifically in CpG islands, where actual gene expression regulation is thought to be more common. Our *in silico* analyses provided guidance by identifying gene promoters most frequently methylated and, most importantly, individual CpG sites significantly anti‐correlated with gene expression (i.e., those in which methylation impaired gene expression). We, thus, selected five genes for validation (including the two indicated in the previous study, *BRCA1* and *RAD51C* [13]) and primer/probe design for qMSP, which we rigorously planned by use of several *in silico* tools, for maximal specificity and capture of relevant CpGs. Subsequent *in silico* analyses of the aggregation of significant CpGs per gene indicated that *BRCA1* and *SYCP3* disclosed, overall, the highest methylation levels, validated by qMSP in our patient cohort. Moreover, methylation levels of the selected genes were significantly higher in NSs, both *in silico* and in our independent cohort. In concordance with our hypothesis, lower expression of these genes was depicted by RNA‐seq data, with significant moderate/strong methylation‐expression anti‐correlations, confirming the report of Shen *et al*. [13] for *RAD51C*. This anti‐correlation between methylation and expression levels was further validated in matched tissues of our own patients' cohort. SE components depicted low methylation levels [62], and in only two pure SE patients *RAD51C* methylation could be detected, although at very minute levels. Shen *et al*. [13] reported *BRCA1* and *RAD51C* hypermethylation (450k array) only in NSs, and particularly in 35% of non‐EC NS cases. Some differences in methodology may explain differences in proportion of cases with detectable hypermethylation (for instance histological categorization of samples, especially of mixed tumors and their individual components, only performed in our study; and definition of gene promoter and consideration of CpGs located exclusively in CpG islands). Nonetheless, our *in silico* analyses and validation studies were consistent with these results, showing higher methylation levels in more differentiated TGCT subtypes, especially YST and TE, compatible with the increasing methylation profile dependent on differentiation [63]. Interestingly, significantly higher methylation levels of *RAD51C* in M+ and intermediate/poor IGCCCG prognosis disease and higher methylation levels of *BRCA1* in stage II/III patients was observed, suggesting association with disease burden.

*BRCA1* methylation, a well‐known major player in HR DNA repair pathway [64], has already been documented in TGCTs by Koul *et al*. [65], which found hypermethylation of genes involved in DNA repair (*RASSF1A*, *BRCA1* and *MGMT*) in 60% of NSs, with lower/absent methylation in SE samples. In that report, *BRCA1* hypermethylation was observed in 20% of TGCTs. This is due to several differences in methodology, namely the use of primers for different *BRCA1* promoter regions (which may also explain why authors did not find a consistent anti‐correlation with gene expression) and the use of methylation‐specific PCR scored in agarose gel, whereas we designed primers and probes to match the probes analyzed in 450k array and used a quantitative method (qMSP, run with standard curves for 45 cycles). *PALB2* is fundamental for the HR pathway by bringing together the BRCA1‐PALB2‐BRCA2‐RAD51 complex [66]. Despite the strong gene expression‐methylation anti‐correlation depicted for this gene *in silico*, in our cohort no methylation was found for this gene. However, this is compatible with the extremely low beta‐values observed in the 450k array (not distinguishable using qMSP). *RAD51C* is another major player of the pathway, and has been identified as a susceptibility loci for TGCT formation [67], again supporting the relevance of the HR pathway in this tumor model. *RAD54* is also relevant, acting in the late phase of HR, namely branch migration of Holiday junctions [68], and *RAD54B*‐deficient tumors were found to benefit from PARPis *in vitro* [69]. Our main hypothesis was that HR genes methylation associated with response to Olaparib, and hence could be used as predictive biomarkers in the clinic. Indeed, we confirmed that Olaparib significantly reduced cell viability and colony formation in EC‐derived TGCT cell lines, supporting the data of Cavallo *et al*. [14], and found that the two most sensitive cell lines were also the ones showing the highest *BRCA1* methylation levels and the only disclosing *RAD51C* methylation (and, additionally, they showed significantly lower expression of both genes compared to TCam‐2). The very recent and important phase II clinical trial by De Giorgi and collaborators demonstrated only marginal activity of Olaparib in the treatment of heavily cisplatin pre‐treated and refractory patients; however, the study identified a case of stable disease that remarkably was the only patient with a BRCA mutation (hence, with impairment of the HR pathway) [70]. We believe this reinforces our data that patients should be optimally selected for these targeted treatments to obtain maximal benefit, as the authors of the trial conclude, and methylation biomarkers could be such key factors for tailoring treatment. Authors also reported five cases of Grade 3–4 adverse events; indeed, evaluation of toxicity on normal HR proficient cells should also be taken into account as a form of toxicity of the drug, given the recent findings of Ito and co‐workers who described increased sister chromatid exchanges and chromatid aberrations in normal cells. *In vivo* studies may be key to evaluate such effects in more detail, and determine the least toxic concentrations [71].

As for *SYCP3*, its role in meiosis and germ cell development is well‐established, being frequently interpreted as a marker of meiotic progression in germ cell tumors [72]. Furthermore, *SYCP3* expression demonstrated prognostic value in other (somatic) tumors [73]. Importantly, Hosoya *et al*. [40] also showed that *SYCP3* impaired HR by interfering with *BRCA2*, ultimately impeding the recruitment of *RAD51* to the double‐stranded breaks in mitotic cells. Moreover, *SYCP3* expression was correlated with increased sensitivity to Olaparib in several somatic tumor cell lines. Since we found a very strong methylation‐expression anti‐correlation for this gene and Hosoya *et al*. [40] reported induced expression after treatment with demethylating agent 5‐aza, we hypothesized that *SYCP3* promoter methylation might serve as biomarker of resistance/lower response to Olaparib treatment. *In silico* findings for this gene were validated in our cohort, with SEs showing the lowest methylation levels and the highest immunoexpression scores. However, immunoexpression was not observed in cell lines and, importantly, the pattern of methylation in cell lines was not informative of response to Olaparib. We then hypothesize that *SYCP3* expression in TGCTs (which was overall low, occurring in few disperse cells within the tumor) may solely represent an aberrant activation of the meiotic program in certain malignant germ cells, which show (failed) attempts to enter meiosis [as previously suggested [74]], and not an indication of BRCA2‐RAD51 axis impairment, although this should be confirmed in further studies.

## 5 Conclusions

Overall, we have demonstrated and validated the rationale for using HR genes methylation, especially *BRCA1* and *RAD51C*, as biomarkers of sensitivity to Olaparib, in a combined *in silico*, *in vitro,* and clinical study. Despite low representation of more infrequent NS subtypes and mixing of all tumor components within TCGA cohort approach, we have surpassed this by dissecting individual tumor components within mixed tumors in our own tissue cohort, assuring representation of all histologies. Moreover, low representation of patients with metastatic events (N+ and M+ disease) precludes robust investigation of these markers in the prognosis especially when stratifying by histological subtypes. Larger studies enriched in poor outcome patients will be instrumental to address this question, which was not the main purpose of the present work. In the future, we intend to validate a multiplex test for these targets in cell‐free DNA in liquid biopsies, to serve as a noninvasive predictive biomarker of response to PARPis. The test also has the potential to identify individuals with tumors depicting impaired HR DNA repair, which may also benefit from cisplatin‐based chemotherapy, allowing for better tailoring of treatment strategies in TGCT patients.

## Conflict of interest

The authors declare no conflict of interest.

## Author contributions

JL and VC performed molecular analyses and wrote the manuscript. JL collected the clinical data and reviewed histological specimens. VC and PL‐S performed in silico analyses. CG‐T and VM‐G assisted in specific tasks related to cell culture, JPS related to immunohistochemistry, and LP related to sample preparation. RG and MC prepared the histological sections for immunohistochemistry. JL assessed the immunohistochemistry. JL and VC analyzed the data. IB and JM provided clinical information about the patients. LL provided the cell lines for the study. RH and CJ supervised the work and revised the manuscript. All authors read and approved the manuscript.

## 6 Ethics approval and consent to participate

This study was approved by the Ethics Committee (CES‐IPO‐1/2018) of Portuguese Oncology Institute of Porto, Portugal. All procedures performed in tasks involving human participants were in accordance with the ethical standards of the institutional and/or national research committee and with the 1964 Helsinki declaration and its later amendments or comparable ethical standards.

## Supporting information


**Fig. S1.** Individual CpG expression‐methylation anti‐correlations for BRCA1. Methylation levels are reported as beta‐values (450k array) and gene expression as Log2‐scaled (TPM+1) (RNA‐sequencing) values. See text for details.

**Fig. S2.** Individual CpG expression‐methylation anti‐correlations for PALB2 and RAD51C. Methylation levels are reported as beta‐values (450k array) and gene expression as Log2‐scaled (TPM+1) (RNA‐sequencing) values. See text for details.

**Fig. S3.** Individual CpG expression‐methylation anti‐correlations for RAD54B and SYCP3. Methylation levels are reported as beta‐values (450k array) and gene expression as Log2‐scaled (TPM+1) (RNA‐sequencing) values. See text for details.

**Fig. S4.** Methylation and expression levels of target genes among histological subtypes. Methylation/expression of BRCA1 (A and B), PALB2 (C and D), RAD51C (E and F), RAD54B (G and H) and SYCP3 (I and J) among the various histological subtypes. Methylation levels are reported as beta‐values (450k array) and gene expression as Log2‐scaled (TPM+1) (RNA‐sequencing) values. Error bars indicate median an interquartile range. Statistical test was Mann–Whitney/Kruskal–Wallis. See text for details. Abbreviations: SE—seminoma; EC—embryonal carcinoma; Mix—mixed tumor; YST—yolk sac tumor; TE—teratoma; TPM—transcript per million.

**Fig. S5.** Differential methylation levels of target genes related to disease burden. Differential methylation of BRCA1, PALB2, RAD51C, RAD54B and SYCP3 between N0 and N+ disease (A) and M0 and M+ disease (B). Methylation levels are reported as beta‐values (450k array). Error bars indicate median an interquartile range. Statistical test was Mann–Whitney. See text for details.

**Fig. S6.** Differential expression levels of BRCA1 and RAD51C (RT‐qPCR validation). Differential expression of BRCA1 (A and C) and of RAD51C (B and D) among seminomas and nonseminomas, and between individual tumor subtypes, respectively. Expression‐methylation anti‐correlation for BRCA1 (E) and RAD51C (F). Expression results are normalized to GUSB. Error bars indicate median an interquartile range. Statistical test was Mann–Whitney/Kruskal–Wallis. Shaded gray area relates to error, set to 95% confidence. Abbreviations: SE—seminoma; NS—nonseminoma; EC—embryonal carcinoma; YST—yolk sac tumor; CH—choriocarcinoma; TE—teratoma.

**Fig. S7.** Differential methylation levels related to disease burden (qMSP validation). Differential methylation of BRCA1 in stage I versus stage II/III disease (A) and of RAD51C in Good versus Intermediate/Poor IGCCCG prognosis disease (B). Results are normalized to ACTB. Error bars indicate median an interquartile range. Statistical test was Mann–Whitney.

**Fig. S8.** SYCP3 gene expression in normal testis and testicular germ cell tumors. A—gene expression levels across several normal tissues and tumor models, highlighting the upregulation in TGCTs (data extracted from GEPIA, see text for details); B—protein expression levels across several normal tissues, highlighting the consistent sole expression in normal testis parenchyma (data extracted from Human Protein Atlas, see text for details).

**Fig. S9.** SYCP3 immunoexpression in TGCT tissue cohort. A—Strong nuclear immunoexpression in spermatocytes of normal testis (positive control) (200x magnification); B‐E—Absence of immunoexpression of SYCP3 in TGCT cell lines (TCam‐2, NCCIT, NTera‐2 and 2102Ep, respectively, 200x magnification); F and G—Two examples of seminomas with high immunoexpression score for SYCP3 (200x and 400x magnification, respectively); H—Example of pure embryonal carcinoma with high immunoexpression score for SYCP3 (400x magnification); I—Example of postpubertal‐type yolk sac tumor with immunoexpression of SYCP3 (400x magnification); J—SYCP3 immunoexpression score among seminoma and nonseminoma tumor components; K—SYCP3 positivity among the various testicular germ cell tumor subtypes. Abbreviations: SE—seminoma; NS—nonseminoma; EC—embryonal carcinoma; CH—choriocarcinoma; YST—yolk sac tumor; TE—teratoma.

**Fig. S10.** Differential expression levels of BRCA1 (A) and RAD51C (B) in cell lines. Results are normalized to GUSB. Error bars indicate median an interquartile range. Statistical test was Kruskal–Wallis.

**Table S1.** Clinicopathological features of the study cohort.

**Table S2.** Primer and probe sequences used in the work.

**Table S3.** Anti‐correlation analyses for individual nonseminoma subtypes.

**Table S4.** Number of metastatic events overall and discriminated per histology.

**File S1.** Primer/probe design for this study.

**File S2.** Summary of *in silico* analysis and CpG site selection for genes implicated in the HR pathway.

## Data Availability

All data generated or analyzed during this study are included in this article and its supplementary information files.
