# Supplementary figures and images for: Promoter methylation of DNA homologous recombination genes is predictive of the responsiveness to PARP inhibitor treatment in testicular germ cell tumors
Source: Mol Oncol. 2021 Mar 2;15(4):846–65. doi: 10.1002/1878-0261.12909 (PMC8024740; doi:10.1002/1878-0261.12909)

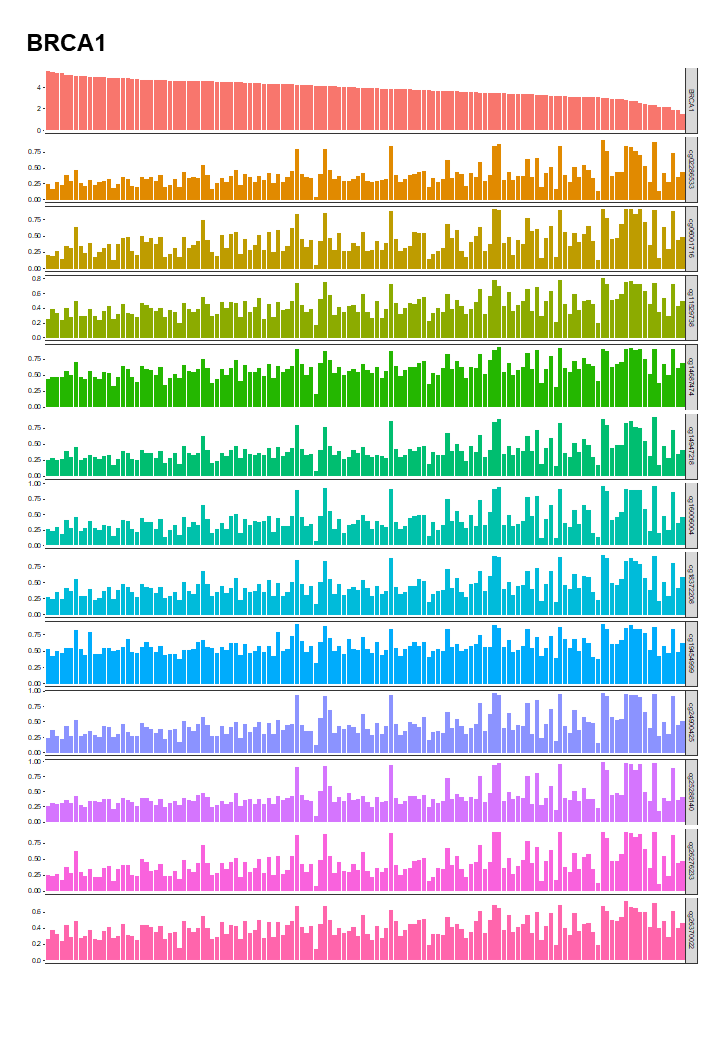

Supplement: Supplementary file 1 — Fig. S1. Individual CpG expression‐methylation anti‐correlations for BRCA1. Methylation levels are reported as beta‐values (450k array) and gene expression as Log2‐scaled (TPM+1) (RNA‐sequencing) values. See text for details. [file MOL2-15-846-s002.tif]

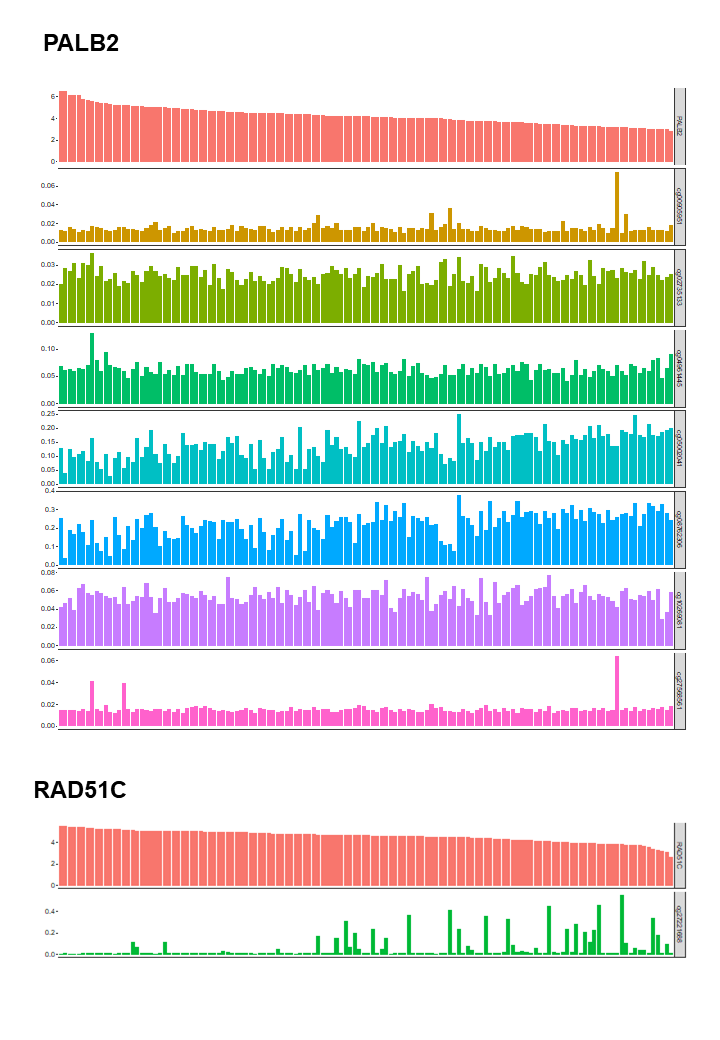

Supplement: Supplementary file 2 — Fig. S2. Individual CpG expression‐methylation anti‐correlations for PALB2 and RAD51C. Methylation levels are reported as beta‐values (450k array) and gene expression as Log2‐scaled (TPM+1) (RNA‐sequencing) values. See text for details. [file MOL2-15-846-s014.tif]

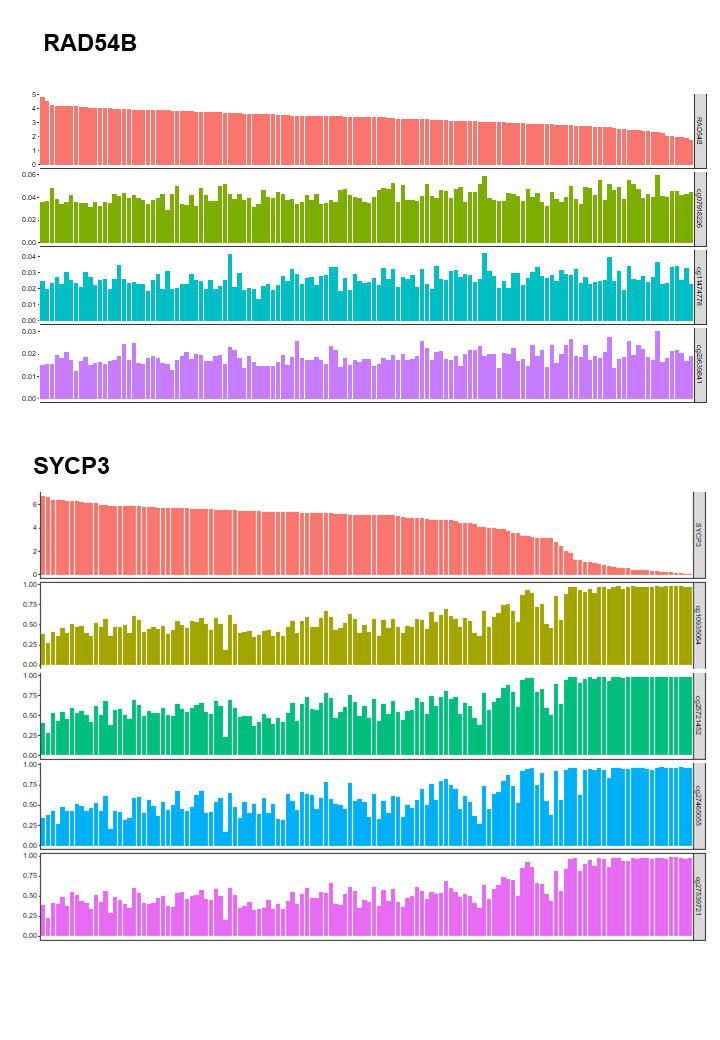

Supplement: Supplementary file 3 — Fig. S3. Individual CpG expression‐methylation anti‐correlations for RAD54B and SYCP3. Methylation levels are reported as beta‐values (450k array) and gene expression as Log2‐scaled (TPM+1) (RNA‐sequencing) values. See text for details. [file MOL2-15-846-s004.tif]

**A**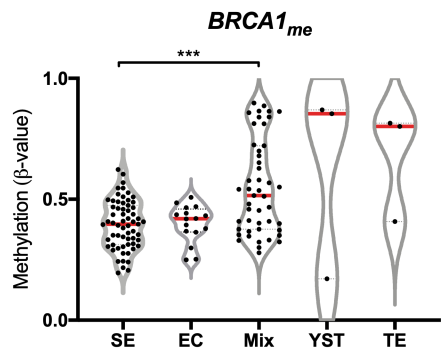**B**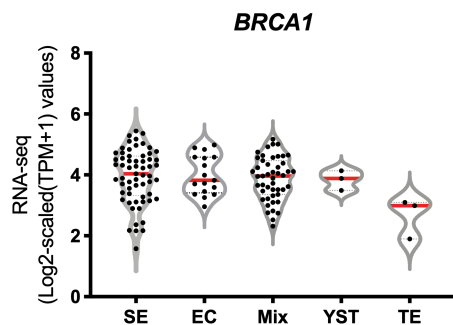**C**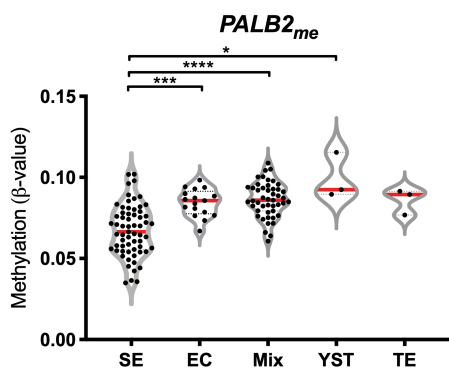**D**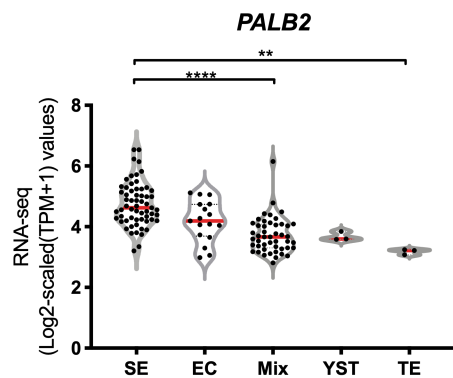**E**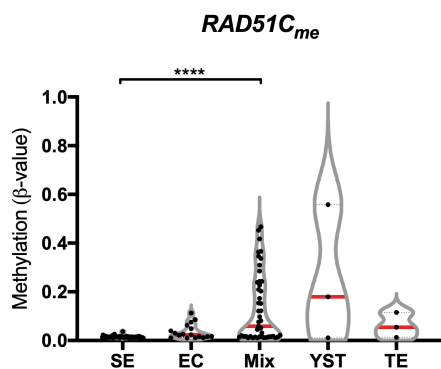**F**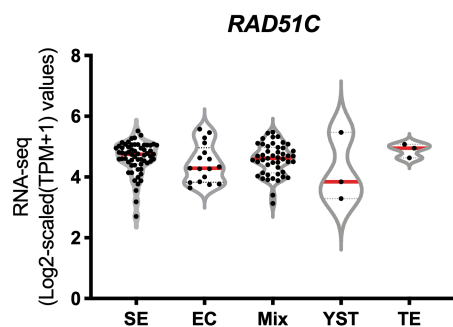**G**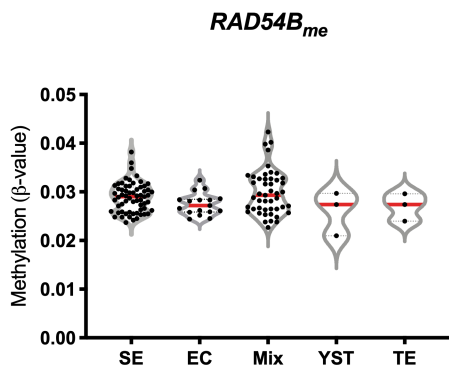**H**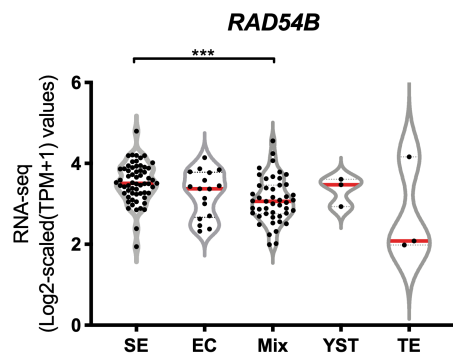**I**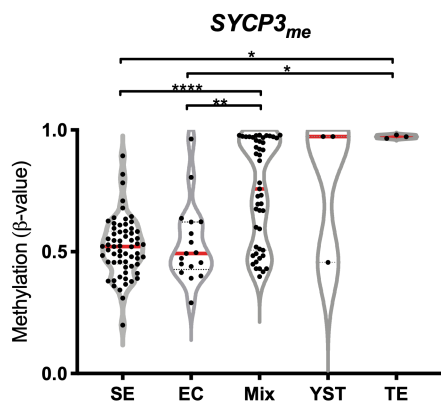**J**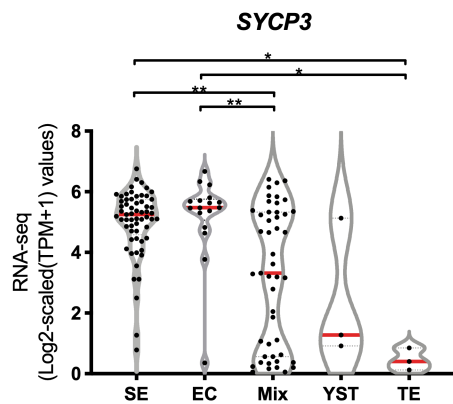

Supplement: Supplementary file 4 — Fig. S4. Methylation and expression levels of target genes among histological subtypes. Methylation/expression of BRCA1 (A and B), PALB2 (C and D), RAD51C (E and F), RAD54B (G and H) and SYCP3 (I and J) among the various histological subtypes. Methylation levels are reported as beta‐values (450k array) and gene expression as Log2‐scaled (TPM+1) (RNA‐sequencing) values. Error bars indicate median an interquartile range. Statistical test was Mann–Whitney/Kruskal–Wallis. See text for details. Abbreviations: SE—seminoma; EC—embryonal carcinoma; Mix—mixed tumor; YST—yolk sac tumor; TE—teratoma; TPM—transcript per million. [file MOL2-15-846-s001.pdf]

# N0 vs N+ disease

# M0 vs M+ disease

BRCA1

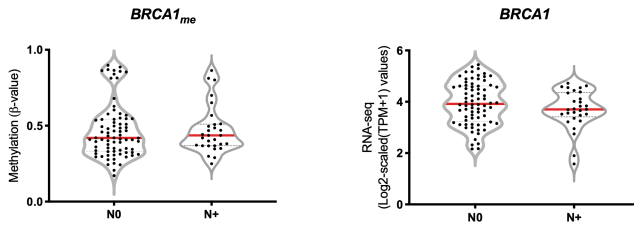

PALB2

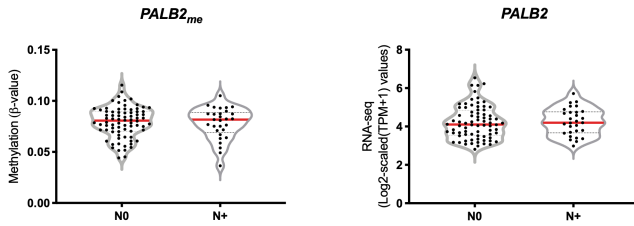

RAD51C

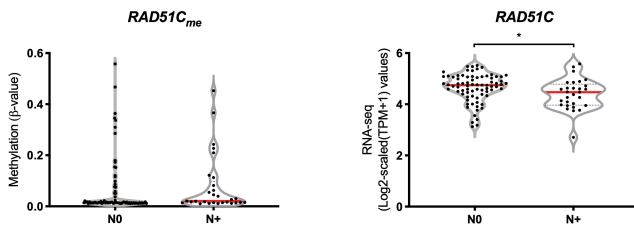

RAD54B

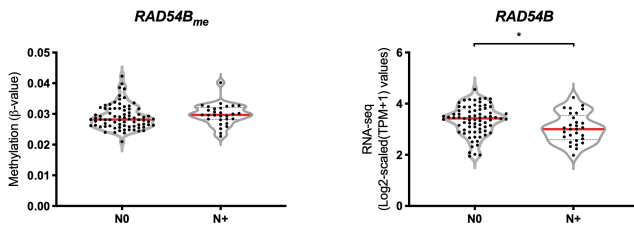

SYCP3

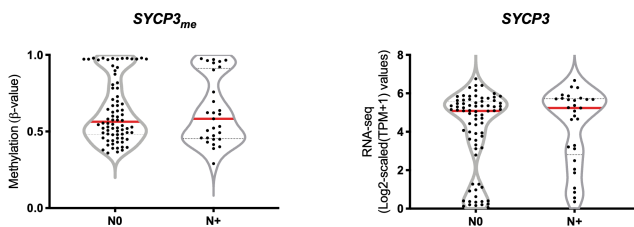

A

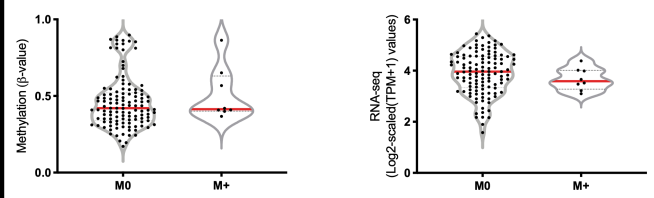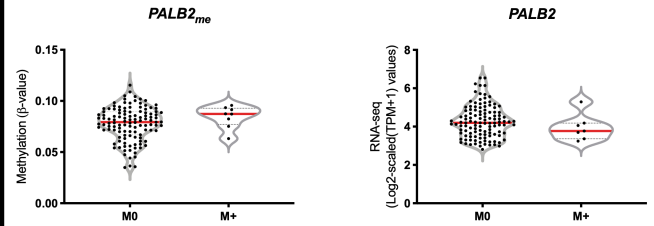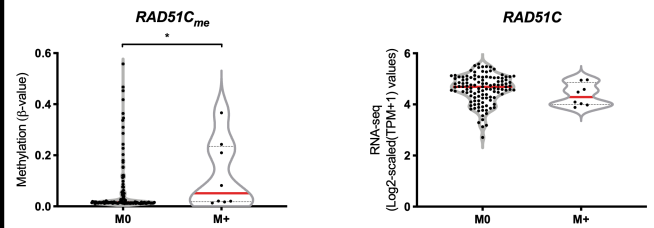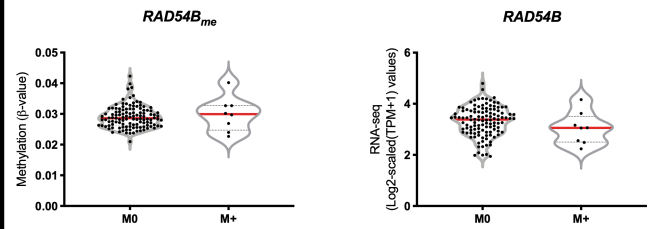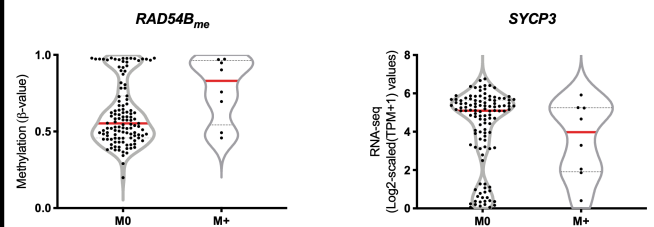

B

Supplement: Supplementary file 5 — Fig. S5. Differential methylation levels of target genes related to disease burden. Differential methylation of BRCA1, PALB2, RAD51C, RAD54B and SYCP3 between N0 and N+ disease (A) and M0 and M+ disease (B). Methylation levels are reported as beta‐values (450k array). Error bars indicate median an interquartile range. Statistical test was Mann–Whitney. See text for details. [file MOL2-15-846-s015.pdf]

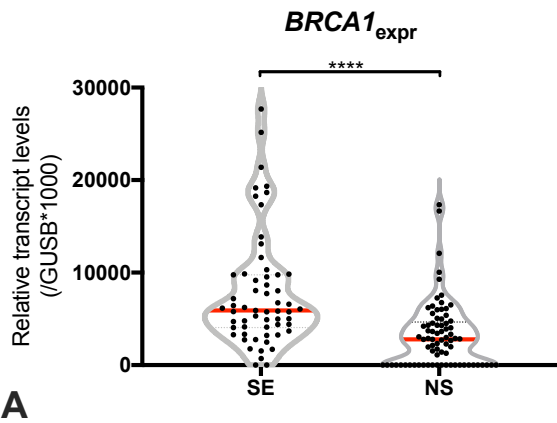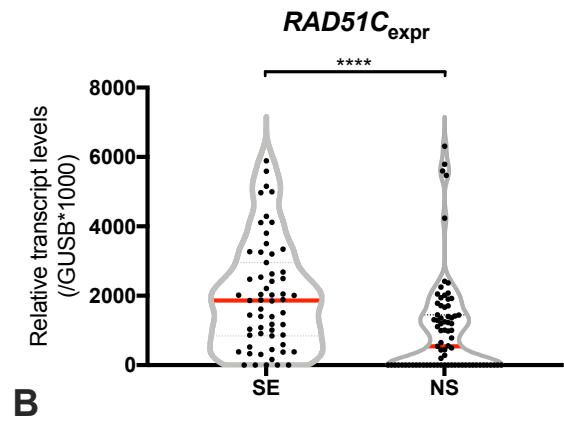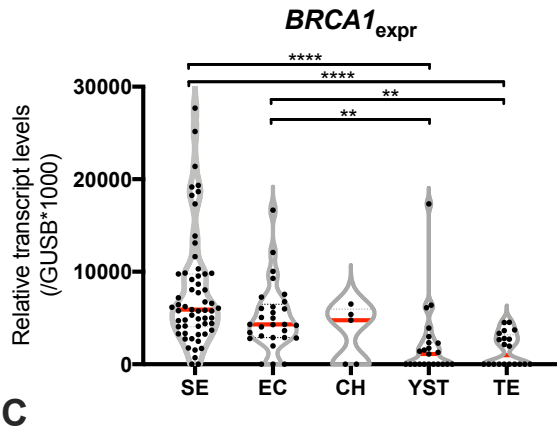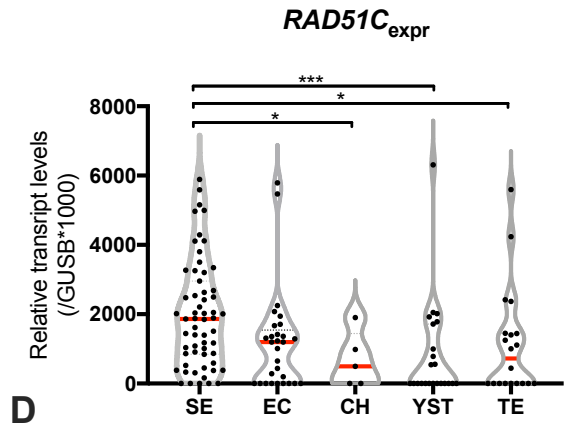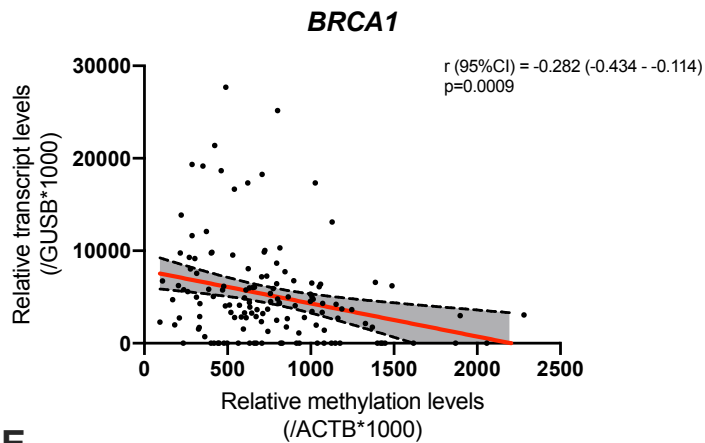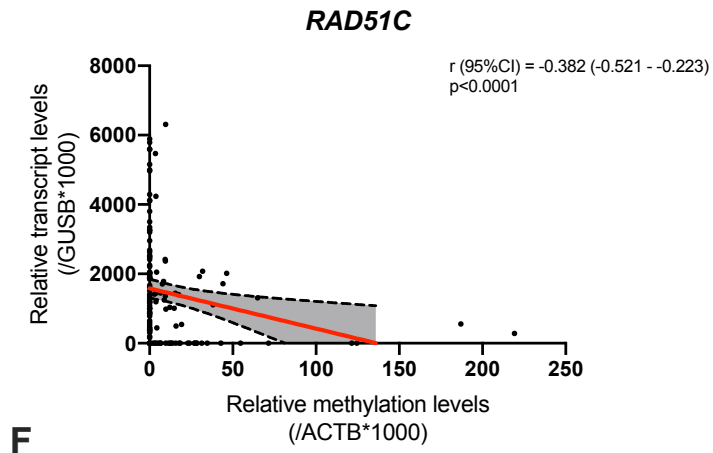

Supplement: Supplementary file 6 — Fig. S6. Differential expression levels of BRCA1 and RAD51C (RT‐qPCR validation). Differential expression of BRCA1 (A and C) and of RAD51C (B and D) among seminomas and nonseminomas, and between individual tumor subtypes, respectively. Expression‐methylation anti‐correlation for BRCA1 (E) and RAD51C (F). Expression results are normalized to GUSB. Error bars indicate median an interquartile range. Statistical test was Mann–Whitney/Kruskal–Wallis. Shaded gray area relates to error, set to 95% confidence. Abbreviations: SE—seminoma; NS—nonseminoma; EC—embryonal carcinoma; YST—yolk sac tumor; CH—choriocarcinoma; TE—teratoma. [file MOL2-15-846-s003.pdf]

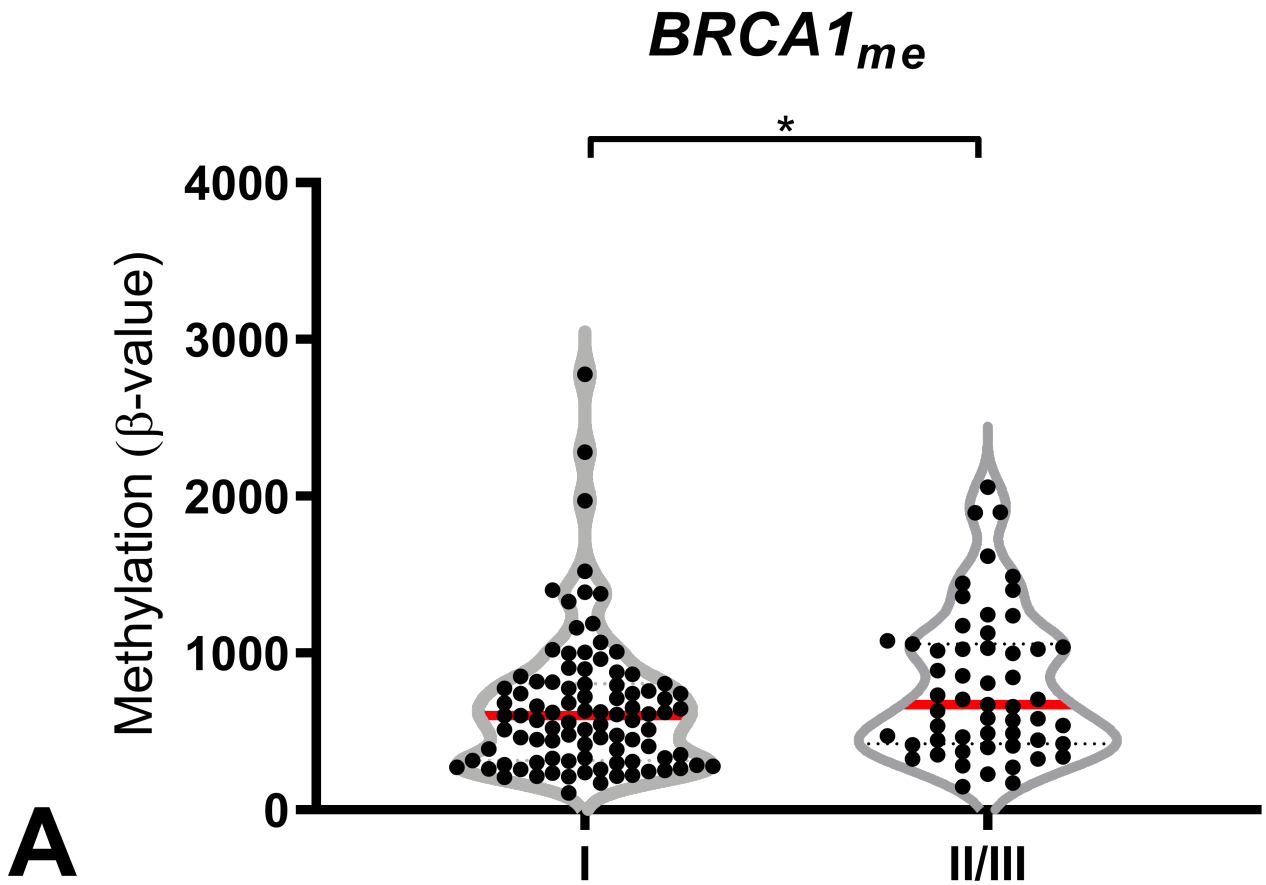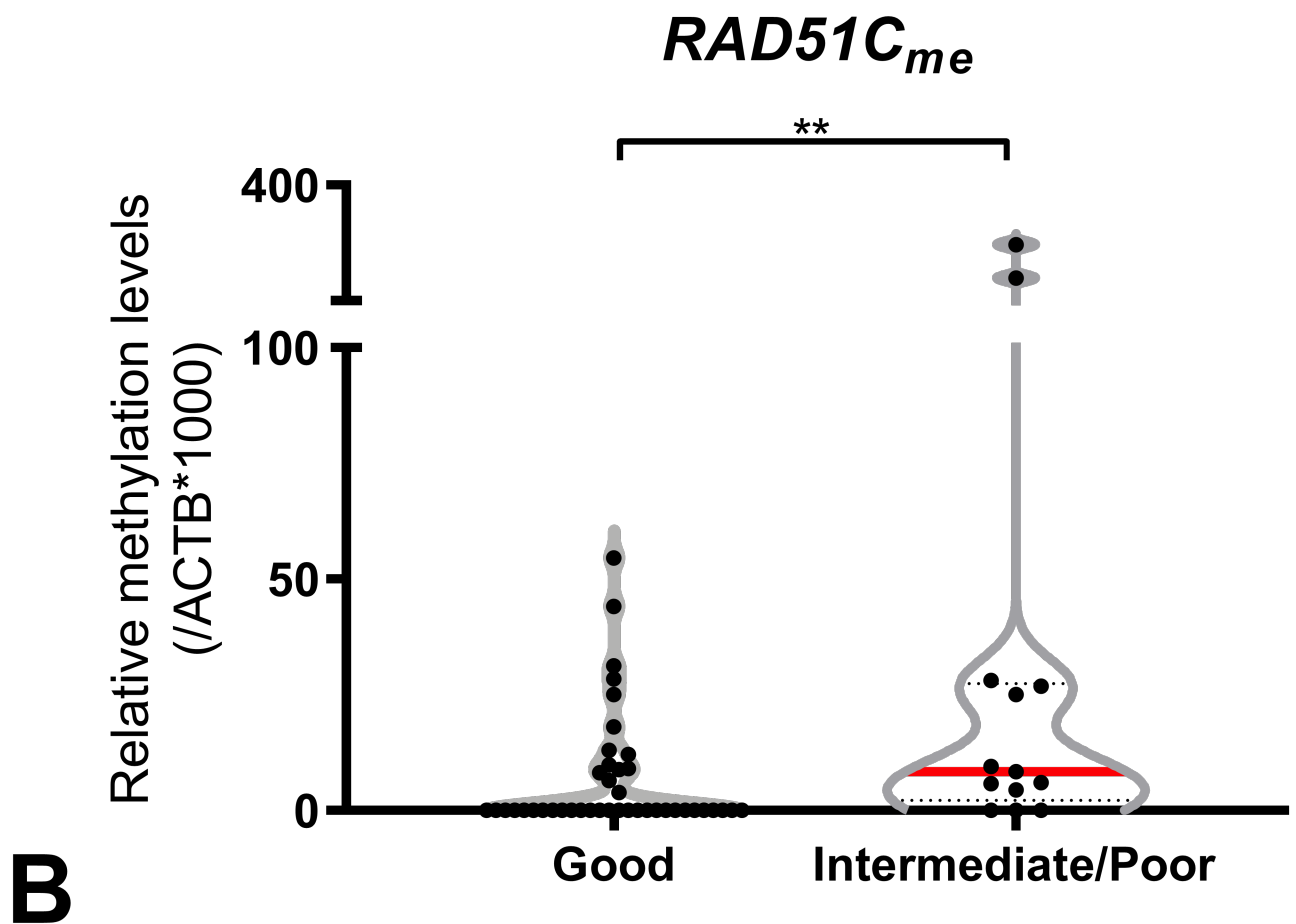

Supplement: Supplementary file 7 — Fig. S7. Differential methylation levels related to disease burden (qMSP validation). Differential methylation of BRCA1 in stage I versus stage II/III disease (A) and of RAD51C in Good versus Intermediate/Poor IGCCCG prognosis disease (B). Results are normalized to ACTB. Error bars indicate median an interquartile range. Statistical test was Mann–Whitney. [file MOL2-15-846-s016.pdf]

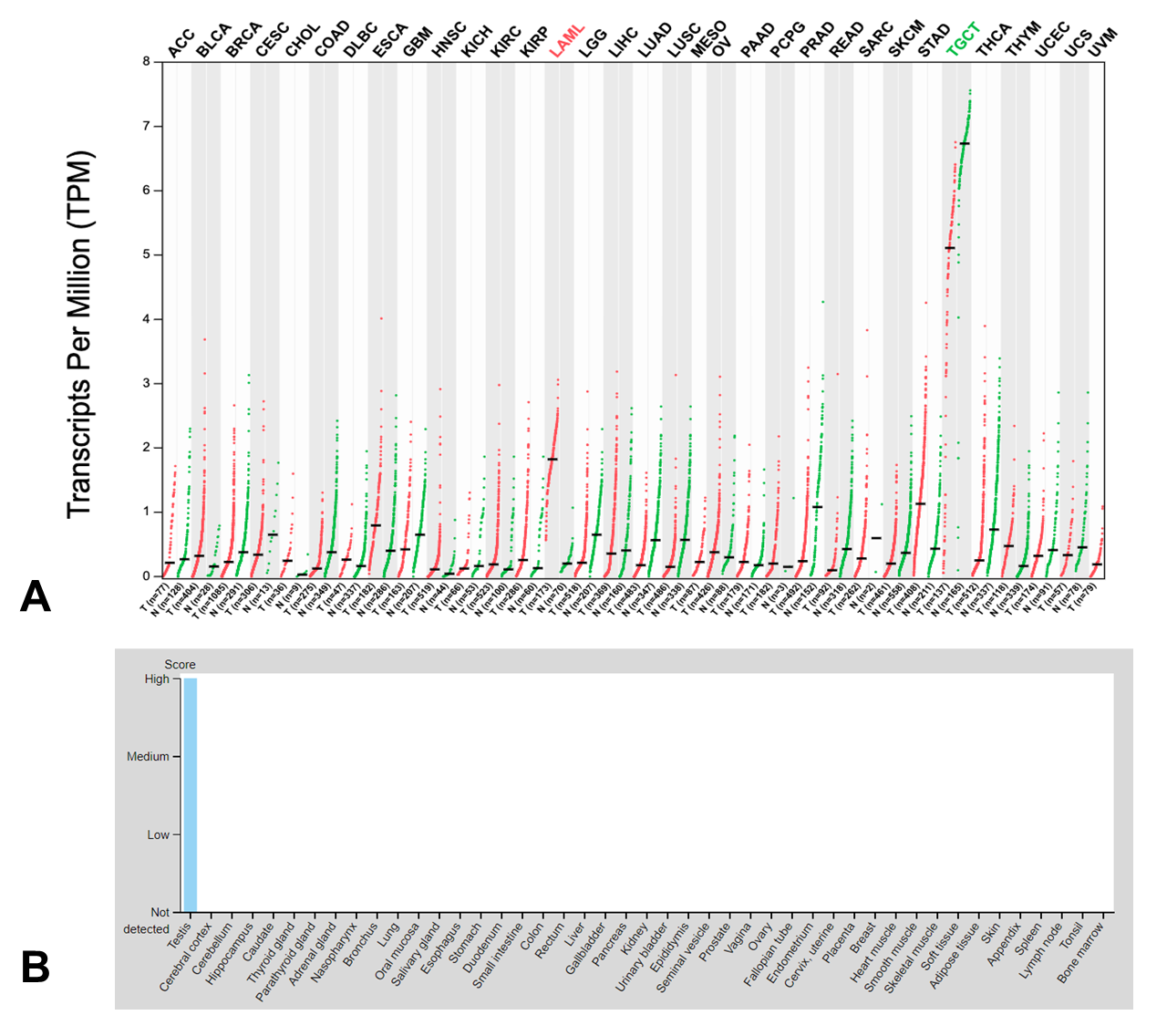

Supplement: Supplementary file 8 — Fig. S8. SYCP3 gene expression in normal testis and testicular germ cell tumors. A—gene expression levels across several normal tissues and tumor models, highlighting the upregulation in TGCTs (data extracted from GEPIA, see text for details); B—protein expression levels across several normal tissues, highlighting the consistent sole expression in normal testis parenchyma (data extracted from Human Protein Atlas, see text for details). [file MOL2-15-846-s008.tif]

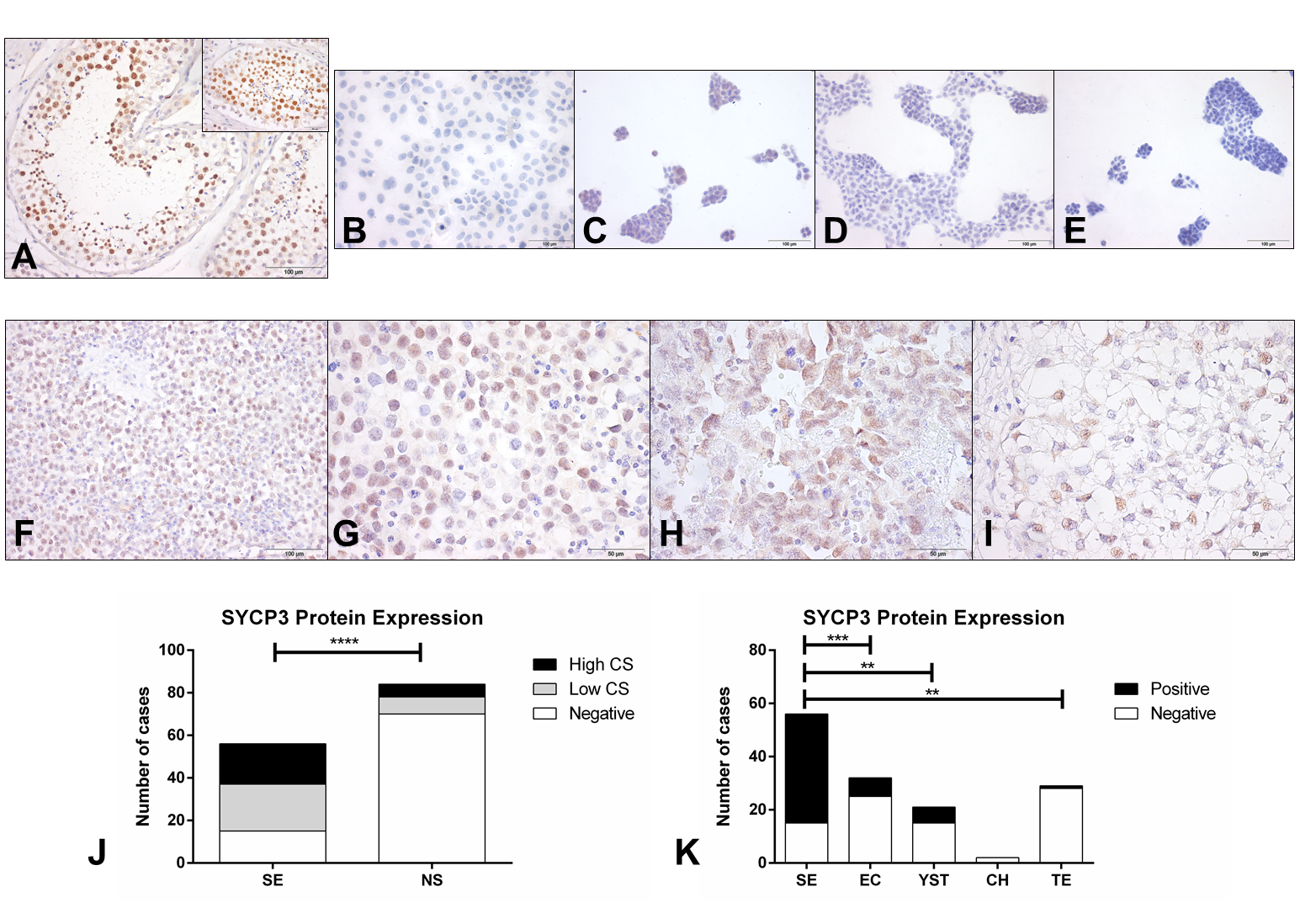

Supplement: Supplementary file 9 — Fig. S9. SYCP3 immunoexpression in TGCT tissue cohort. A—Strong nuclear immunoexpression in spermatocytes of normal testis (positive control) (200x magnification); B‐E—Absence of immunoexpression of SYCP3 in TGCT cell lines (TCam‐2, NCCIT, NTera‐2 and 2102Ep, respectively, 200x magnification); F and G—Two examples of seminomas with high immunoexpression score for SYCP3 (200x and 400x magnification, respectively); H—Example of pure embryonal carcinoma with high immunoexpression score for SYCP3 (400x magnification); I—Example of postpubertal‐type yolk sac tumor with immunoexpression of SYCP3 (400x magnification); J—SYCP3 immunoexpression score among seminoma and nonseminoma tumor components; K—SYCP3 positivity among the various testicular germ cell tumor subtypes. Abbreviations: SE—seminoma; NS—nonseminoma; EC—embryonal carcinoma; CH—choriocarcinoma; YST—yolk sac tumor; TE—teratoma. [file MOL2-15-846-s009.tif]

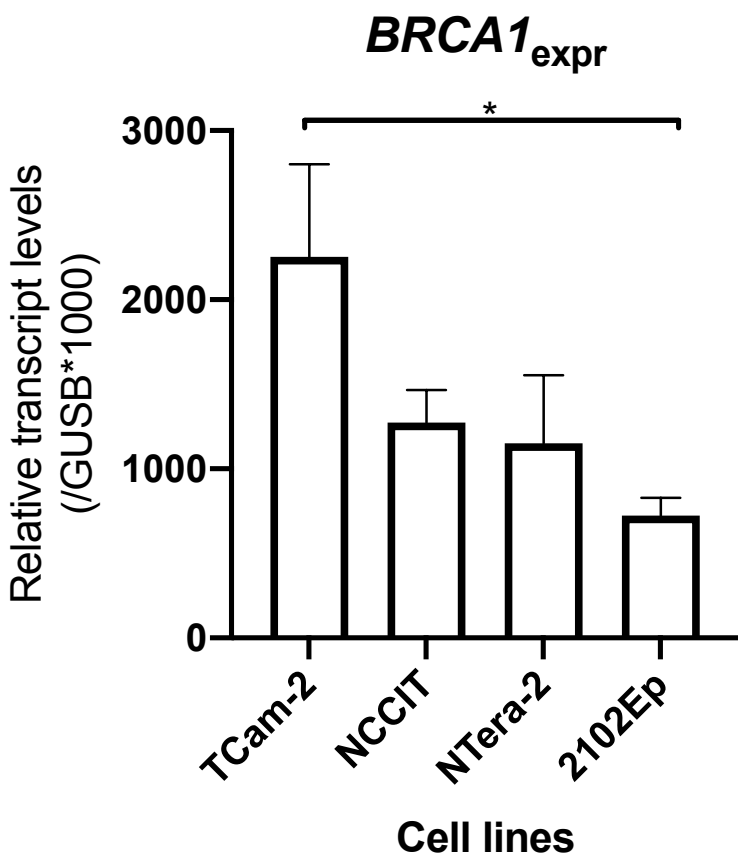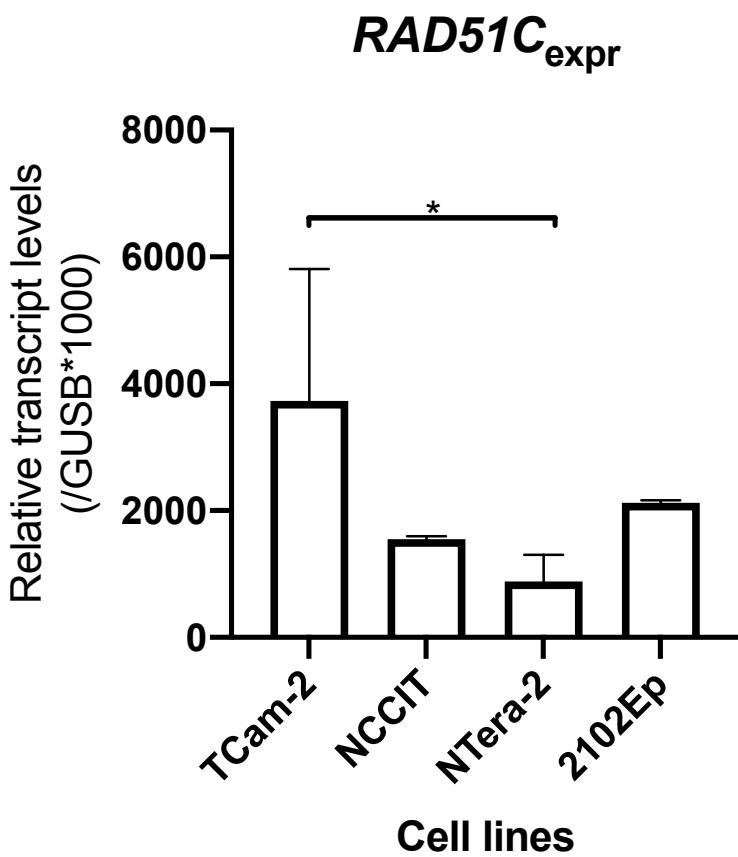

Supplement: Supplementary file 10 — Fig. S10. Differential expression levels of BRCA1 (A) and RAD51C (B) in cell lines. Results are normalized to GUSB. Error bars indicate median an interquartile range. Statistical test was Kruskal–Wallis. [file MOL2-15-846-s007.pdf]
