# Supplementary material for: Promoter methylation of DNA homologous recombination genes is predictive of the responsiveness to PARP inhibitor treatment in testicular germ cell tumors
Source: Mol Oncol. 2021 Mar 2;15(4):846–65. doi: 10.1002/1878-0261.12909 (PMC8024740; doi:10.1002/1878-0261.12909)
Supplement: Supplementary file 11 — Table S1. Clinicopathological features of the study cohort. [file MOL2-15-846-s006.docx]

**Supplementary Table 1. Clinicopathological features of the study cohort.**

| Variables | Primary TGCT cases |
| --- | --- |
| Age (median, interquartile range) | 30 (25-36) |
| Histologic subtypes – TGCT patients (n, %) |  |
| Pure seminoma | 82/150 (54.7) |
| Pure embryonal carcinoma | 10/150 (6.7) |
| Pure postpubertal-type teratoma | 1/150 (0.6) |
| Mixed tumor | 57/150 (38.0) |
| Histological subtypes – individual components (n, %) |  |
| Seminoma | 103/238 (43.3) |
| Embryonal carcinoma | 54/238 (22.7) |
| Postpubertal-type yolk sac tumor | 34/238 (14.3) |
| Choriocarcinoma | 10/238 (4.2) |
| Postpubertal-type teratoma | 37/238 (15.5) |
| Stage (n, %) |  |
| I | 95/150 (63.3) |
| II | 32/150 (21.3) |
| III | 23/150 (15.4) |
| IGCCCG Prognostic Group, for metastatic patients (n, %) |  |
| Good | 42/55 (76.4) |
| Intermediate | 6/55 (10.9) |
| Poor | 7/55 (12.7) |
| Relapse |  |
| No | 141/150 (94.0) |
| Yes | 9/150 (6.0) |

Abbreviations: TGCT – testicular germ cell tumors; IGCCCG – International Germ Cell Cancer Collaborative Group
