# Supplementary material for: Promoter methylation of DNA homologous recombination genes is predictive of the responsiveness to PARP inhibitor treatment in testicular germ cell tumors
Source: Mol Oncol. 2021 Mar 2;15(4):846–65. doi: 10.1002/1878-0261.12909 (PMC8024740; doi:10.1002/1878-0261.12909)
Supplement: Supplementary file 12 — Table S2. Primer and probe sequences used in the work. [file MOL2-15-846-s011.docx]

**Supplementary Table 2: Primer and probe sequences used in the work**

| **Gene** | **Primer sequence**  **(5’-3’)** | **Primers, volume**  **(μL), F+R** | **Probe, volume (μL)** | **Annealing**  **Temperature**  **(°C)** |
| --- | --- | --- | --- | --- |
| BRCA1meth | F: GGGAGGCGGTAATGTAAAGATC | 0.4 | 0.05 | 60ºC |
|  | R: CAATCTTCTTAACGAAAACGCG |  |  |  |
|  | Probe: CCCCACAAAAATAACGACAAAACTAACAACG (FAM-BHQ1) |  |  |  |
| PALB2meth | F: GAGATTTCGGTTATTTTCGGTCG | 0.4 | 0.05 | 60ºC |
|  | R: GCACGCGCAACCGTAAAC |  |  |  |
|  | Probe: AGGCGGTTTCGTTTTATTGTTCGGTCG (HEX-BHQ1) |  |  |  |
| RAD54Bmeth | F: GTGTAGCGGTTAGGTAGTGCGTAC | 0.4 | 0.05 | 60ºC |
|  | R: ACTATCCCCTCGCCGCC |  |  |  |
|  | Probe: CGAACGAACCCGCCGCGC  (Cy5-BHQ2) |  |  |  |
| RAD51Cmeth | F: AGTTTCGTGCGGTTAGGTCG | 0.4 | 0.05 | 60ºC |
|  | R: GTCTTCCCGCGCATCG |  |  |  |
|  | Probe: CGTTTTAGCGAGGGCGTGCGG (HEX-BHQ2) |  |  |  |
| ACTB | F: TGGTGATGGAGGAGGTTTAGTAAGT | 0.4 | 0.05 | 60ºC |
|  | R: ACCAATAAAACCTACTCCTCCCTTAA |  |  |  |
|  | Probe: ACCACCACCCAACACACAATAACAAACACA (Cy5-BHQ2) |  |  |  |
| SYCP3meth | F: ATTCGTAATTCGTTTCGGCG | 0.2 | 0.05 | 64ºC |
|  | R: CTAAACGCAAAACGCAAACG |  |  |  |
|  | Probe: TGGGTTAGTTATTTGGGCGCGTAATCG (ATTO-BHQ2) |  |  |  |
| BRCA1exp | F: CTGAAGACTGCTCAGGGCTATC | 0.3 | - | 62ºC |
|  | R: AGGGTAGCTGTTAGAAGGCTGG |  |  |  |
| RAD51Cexp | F: GTGAAACCCTCCGAGCTTAGCA | 0.3 | - | 64ºC |
|  | R: CCTGCTCAAGAAGTTCCAGTGC |  |  |  |
| GUSBexp | F: CACTGAAGAGTACCAGAAAAGTC | 0.5 | - | 62ºC |
|  | R: TCTCTGCCGAGTGAAGATCC |  |  |  |
