# Supplementary material for: Promoter methylation of DNA homologous recombination genes is predictive of the responsiveness to PARP inhibitor treatment in testicular germ cell tumors
Source: Mol Oncol. 2021 Mar 2;15(4):846–65. doi: 10.1002/1878-0261.12909 (PMC8024740; doi:10.1002/1878-0261.12909)
Supplement: Supplementary file 13 — Table S3. Anti‐correlation analyses for individual nonseminoma subtypes. [file MOL2-15-846-s013.docx]

**Supplementary Table 3: Anti-correlation analyses for individual non-seminoma subtypes**

| **Genes** | BRCA1exp | PALB2exp | RAD54Bexp | RAD51Cexp | SYCP3exp |
| --- | --- | --- | --- | --- | --- |
| BRCA1meth | Pure EC:  r=-0.689, p=0.002  Mixed Tumors: r=-0.686, <0.0001 |  |  |  |  |
| PALB2meth |  | Pure EC:  r=-0.289, p=0.260  Mixed Tumors: r=-0.479, p=0.001 |  |  |  |
| RAD54Bmeth |  |  | Pure EC:  r=-0.755, p<0.0001  Mixed Tumors:  r=-0.553, p<0.0001 |  |  |
| RAD51Cmeth |  |  |  | Pure EC:  r=-0.654, p=0.004  Mixed Tumors:  r=-0.614, p<0.0001 |  |
| SYCP3meth |  |  |  |  | Pure EC:  r=-0.583, p=0.014  Mixed Tumors:  r=-0.932, p<0.0001 |
