# Supplementary material for: Promoter methylation of DNA homologous recombination genes is predictive of the responsiveness to PARP inhibitor treatment in testicular germ cell tumors
Source: Mol Oncol. 2021 Mar 2;15(4):846–65. doi: 10.1002/1878-0261.12909 (PMC8024740; doi:10.1002/1878-0261.12909)
Supplement: Supplementary file 14 — Table S4. Number of metastatic events overall and discriminated per histology. [file MOL2-15-846-s012.docx]

Supplementary Table 4 – Number of metastatic events overall and discriminated per histology

| Histology | N0 | N+ | M0 | M+ |
| --- | --- | --- | --- | --- |
| Total cases | 78 | 29 | 119 | 8 |
| Only SE | 39 | 8 | 57 | 1 |
| Only NS | 39 | 21 | 62 | 7 |
| Only Mixed Tumors | 28 | 11 | 40 | 6 |
| Only EC | 6 | 9 | 17 | 0 |
| Only YST | 3 | 0 | 3 | 0 |
| Only TE | 2 | 1 | 2 | 1 |
