# Supplementary material for: Promoter methylation of DNA homologous recombination genes is predictive of the responsiveness to PARP inhibitor treatment in testicular germ cell tumors
Source: Mol Oncol. 2021 Mar 2;15(4):846–65. doi: 10.1002/1878-0261.12909 (PMC8024740; doi:10.1002/1878-0261.12909)
Supplement: Supplementary file 15 — File S1. Primer/probe design for this study. [file MOL2-15-846-s010.docx]

PRIMER / PROBE DESIGN*

*BRCA1:*

**TT**TTGAA**T**TT**T**T**TT**AAA**TTT**T**T**TTAGTGTGA**C**GTGA**TTTT**A**TTTT**TAG**T**TAA**TTT**AGG**T**TG**T**TT**TT**TTA**TT**AG**T**TT**TTC**G**TTTTT**TGGGGAGG**C**GG**T**AATG**T**AAAGA**TC**GT**TC**G**T**TG**TT**AG**T**T**T**TG**TC**G**T**TAT**T**T**T**TGTGGGGTGAAT**T**TAA**T**ATGG**C**GGA**T**AAAGA**T**AGTAA**T**TAGT**TTC**GTTT**T**T**TC**G**C**GTTTT**C**G**TT**AAGAAGATTGG**T**T**T**TTA**TT**A**T**TTGT**TTT**T**T**AAAA**C**GA**TT**A**TTTT**ATTGA**T**TGGTGG**C**GATTG**C**GT**C**GA**C**GGAGA**C**GGGG**T**AAAAG**T**AAG**T**TGAA**TTC**GAAAAATAA**T**AAA**T**A**T**TGGGG**T**TGAGGGGTGGAA**T**TA**C**GAGTG**C**G**T**AGA**T**ATGGG**TT**AGAG**C**G**T**ATTT**TTTT**TG**TTTT**AGG**T**AAATT**C**GG**C**G**T**T**T**A**T**TG**C**GT**TTTC**G**T**AGG**TT**A**T**TGA**TT**TTA**T**AAGA**T**TA**T**TTG**TTTT**AGA**T**T**TT**TGGGG**T**TGGATG

*PALB2:*

AGGGG**T**AGGTTGGGAA**C**G**TC**GA**TT**AGG**TT**T**T**AAAGGGA**C**G**T**AGGGTTGGAAAGAGGAGGATA**T**ATAT**T**TGGA**TTC**G**C**G**T**A**TC**GT**T**T**C**GATGTA**T**T**T**AGA**T**TTGTTGTAGAG**T**AG**T**T**C**G**TTT**AA**T**T**TT**ATGG**TC**G**TC**G**TT**T**TT**TT**TTT**T**T**AG**TTTC**GGAT**TT**TGT**T**AGAGT**T**AGT**C**GGT**T**AGT**T**TTT**T**AGAGATT**TC**GG**T**TA**T**TT**TC**GG**TC**G**T**T**TT**T**TT**A**T**TT**TC**G**T**T**TT**AGGTGG**TTT**A**T**TGGGA**T**T**T**AT**C**GA**T**AG**C**G**C**GG**T**T**T**T**TT**TTTAGG**C**GG**TT**T**C**G**T**T**TT**A**T**TG**T**T**C**GG**TC**GT**T**TA**C**GG**T**TG**C**G**C**GTG**C**G**T**AGG**TC**GAATGGTGGATTTAATTGG**TC**GGAGTTTAGGG**C**G**C**G**T**TTGG**TTC**G**C**GTGGGT**T**AG**T**TGAT**C**G**C**G**T**A**T**TGAGGGTG**C**GAT**TTC**GGG**T**T**TTTT**ATT**TT**TT**TT**TGGGG**C**G**TT**T**TT**

*RAD51C:*

**T**TGG**T**T**T**T**TC**GT**TT**TATGGT**T**T**TC**GT**TT**AT**C**GTTTTA**T**AGT**T**AGGGTGTA**T**ATTTGATGAGGAAT**T**T**T**TAAATGGGATTTTGGGGAAT**T**AAAA**C**GGAATGGTG**T**ATAAGTGTGAAAATTTA**T**AAGA**T**TG**C**G**T**AAAG**T**TG**T**AAGG**TTC**GGAG**TTTC**GTG**C**GG**TT**AGG**TC**G**T**AGAG**TC**GG**TTTT**TT**TC**G**T**TTTA**C**GT**T**TGA**C**GT**T**A**C**G**TC**G**T**A**C**G**TTTT**AG**C**GAGGG**C**GTG**C**GGAGTTTGG**T**TG**T**T**TC**GGGGTTAG**T**AGGTGAG**TT**TG**C**GATG**C**G**C**GGGAAGA**C**GTT**TC**G**T**TTTGAAATG**T**AG**C**GGGATTTGGTGAGTTT**TTC**G**T**TGT**T**T**TT**AG**C**GGTG**C**GGGTG

*RAD54B:*

AT**T**TA**T**A**T**TGTGT**T**TGAAAGTAGG**T**A**T**TAAA**TT**AGTATTTTTTTAATTGAA**TC**GTAAA**T**TTGAAT**T**AAA**T**TTGGAAG**T**ATTTAAAG**TT**AAA**C**G**T**AAAGATA**T**ATG**C**G**T**AGTGATG**T**AGTAGGGT**C**GT**TT**AAT**T**AGAGTAGGG**T**AGGA**T**AGGG**T**AGTG**T**AG**C**GG**T**TAGGTAGTG**C**G**T**A**C**G**C**G**C**GG**C**GGG**T**T**C**GT**TC**G**C**GATTGG**T**TT**C**G**TC**GAGG**C**GGGAT**TT**TTGAG**T**TT**T**T**TC**GG**C**GG**C**GAGGGGATAG**T**TGGTTA**TT**AGAAGGA**T**TT**T**TTTG**T**AGGG**TT**AGTGGTTT**T**TGT**T**AGATTTT**C**G**TC**GGT

*SYCP3:*

AGATGGGTTT**C**G**TT**ATGTTGGA**T**AGG**T**TGGT**T**TGGAA**T**T**TT**TGA**TT**T**T**AAGTGAT**TC**GT**T**TG**TT**T**C**GG**TT**T**TTT**AAAGTG**T**TGGGATTA**T**AGGTGTGAG**TT**A**T**TG**C**G**TT**TAG**T**TG**T**AATTTT**C**GATTATAAA**T**ATTTAAAA**T**TTAAAAA**T**TGGAAGG**T**A**TT**AGG**TT**T**T**AAGTGTA**T**T**C**G**T**AA**TTC**G**T**T**TC**GG**C**GG**TT**AAAG**T**A**TTT**TGGG**TT**AG**T**TATTTGGG**C**G**C**G**T**AAT**C**G**TTTT**ATGGGGG**C**GGAG**TT**T**C**G**TT**A**TTT**AGTT**T**A**C**G**TT**TG**C**G**T**T**T**TG**C**GT**TT**AG**TT**AAT**T**AG**C**GA**C**GTGT**TC**GGATGAGG**C**GGGA**T**TGG**T**TTTT**T**T**TT**TGTG**C**GAAT**TTT**AG

*Legends:

Yellow “C”: methylated cytosine

Pink “T”: unmethylated cytosine, converted to uracil, detected as thymine

Red “C”: methylated cytosine of interest, as determined by *in silico* analyses

Green highlight: forward primer

Blue highlight: reverse primer

Grey highlight: probe
