# Supplementary material for: Promoter methylation of DNA homologous recombination genes is predictive of the responsiveness to PARP inhibitor treatment in testicular germ cell tumors
Source: Mol Oncol. 2021 Mar 2;15(4):846–65. doi: 10.1002/1878-0261.12909 (PMC8024740; doi:10.1002/1878-0261.12909)
Supplement: Supplementary file 16 — File S2. Summary of in silico analysis and CpG site selection for genes implicated in the HR pathway. [file MOL2-15-846-s005.pdf]

Overview of the analysis

			Gene expression-methylation anti-correlation	
Gene	CpG total	Number of CpGs in Islands&TSS200/1500 (promoter)	Sig <0.05 (number of GpGs)	Aggregation of CpGs
ATM	53	15	2	n.s.
BRCA1	48	12	12	sig.
RAD52	37	6	4	sig.
MUS81	33	11	5	sig.
RPA1	26	1	0	n.a.
SSBP1	19	4	2	n.s.
NBN	19	2	0	n.s.
PALB2	18	7	2	n.s.
UIMC1	18	3	0	n.s.
RBBP8	16	9	1	n.s.
RPA2	16	9	7	sig.
BLM	16	6	0	n.s.
BARD1	15	5	2	n.s.
BRCA2	15	2	0	n.s.
RAD54B	14	3	3	sig.
EME1	13	5	1	sig.
RAD51C	13	1	1	n.a.
BRIP1	10	5	1	n.s.
RAD51D	10	4	1	n.s.
FANCD2	10	2	0	n.s.
BRCC3	9	4	1	n.s.
SYCP3	7	4	4	sig.

Legends: sig = significant; n.s. = non-significant; n.a. not applicable
